# Measuring skill via player dynamics in football dribbling

**DOI:** 10.1038/s41598-023-45914-6

**Published:** 2023-11-03

**Authors:** Lukas Brink, Seung Kyun Ha, Jack Snowdon, Ferran Vidal-Codina, Bobby Rauch, Fan Wang, David Wu, Maurici A. López-Felip, Christophe Clanet, Anette E. Hosoi

**Affiliations:** 1 Department of Aeronautics and Astronautics, Massachusetts Institute of Technology, Cambridge, MA 02139 USA; 2 Department of Chemical Engineering, Massachusetts Institute of Technology, Cambridge, MA 02139 USA; 3 Department of Electrical Engineering and Computer Science, Massachusetts Institute of Technology, Cambridge, MA 02139 USA; 4 Department of Mechanical Engineering, Massachusetts Institute of Technology, Cambridge, MA 02139 USA; 5 Department of Civil and Environmental Engineering, Massachusetts Institute of Technology, Cambridge, MA 02139 USA; 6 Kognia Sports Intelligence, Barcelona, Spain; 7 Center for the Ecological Study of Perception and Action, Department of Psychological Sciences, University of Connecticut, Storrs, CT 06269 USA; 8 Laboratoire d’Hydrodynamique de l’École Polytechnique (LadHyX), Palaiseau, France

**Keywords:** Engineering, Applied mathematics, Scientific data

## Abstract

Although a myriad of studies have been conducted on player behavior in football, in-depth studies with structured theory are rare due to the difficulty in quantifying individual player skills and team strategies. We propose a physics-based mathematical model that describes football players’ movements during dribbling situations, parameterized by the attacker aggressiveness, the defender hesitance and the top speed of both players. These player- and situation-specific parameters are extracted by fitting the model to real player trajectories from Major League Soccer games, and enable the quantification of player dribbling attributes and decisions beyond classical statistics. We show that the model captures the essential dribbling dynamics, and analyze how differences between parameters in varying game situations provide valuable insights into players’ behavior. Lastly, we quantitatively study how changes in the player’s parameters impact dribbling performance, enabling the model to provide scientific guidance to player training, scouting and game strategy development.

## Introduction

The sport of association football, also known as “football” or “soccer”, is one of the most popular sports in the world, attracting over 3 billion fans globally. In this sport, the dynamics of two coupled fluid collectives of individuals (i.e., twenty-two players on two teams) and its higher-order invariants engendered lawfully by the spatial layout (i.e., playing area), as well as rule-based contexts, define one of the richest settings that one can encounter to study human behavior. The game in its natural form requires high precision both at the individual level and at the level of team coordination dynamics, which define a team’s playing style. A variety of different playing styles through which players and teams can excel have been recognized and studied^1–5^. However, despite style peculiarities visible to the eyes of the fans, the fundamental principles of the game that allow players and teams with such different styles to flourish are not so apparent. Thus, looking at the principles underlying the game requires in-depth scientific study. Given coaches’ thorough knowledge of different playing styles, this will require, first and foremost, a theory of the game that allows scientific inquiry to be guided in a common framework by coaching insights and questions.

Traditionally, however, the wide variety of scientific studies related to football have investigated isolated levels of analysis of the game. These levels vary from physiology^6,7^ to biomechanics^8,9^ or players’ perceptual and cognitive skills^10–12^ all the way to statistical and mathematical methods^13–18^.

Although these studies provide valuable contributions towards a scientific understanding of football, they do not seek to understand the game from a unified scientific and coaching perspective from which research inquires may be guided. A unified theory-driven and practice-oriented framework, a kind of a functional (situational) semantics, has been previously advocated for sports in general^19^ and more specifically for football^20^. Indeed, a functional semantics approach is a hallmark of ecological psychology^21^. In football, this must account for the innumerable and dependent interactions in the game to describe the environment “for-the-organism”^22,23^, namely, the players, and the dynamic actions of the organism commensurate with that environment^24,25^.

It has been contended that, to achieve a level of analysis which unifies coaching and scientific insights—which is therefore dependent on “when”, “where” and “who” explaining the interdependent, relational and situational game dynamics^26^—should necessarily contain a common point of reference, an invariant that remains the same across a game^27^. The ball is such an element: At any instance of the game, any slice of time representing a moment of the game, the ball can provide the minimum context to start building knowledge of the specific game situation. Independently of whether one is a football coach teaching the fundamental tactics of the game or a scientist trying to predict the driving parameters by which players self-organize, the ball is paramount for a sound theoretical framework from which one may define playing styles (as a coach) or determine predictive models (as a scientist). The ball’s position, thus, constitutes the fundamental predictor for the behavior of the players and team dynamics at large.

In this paper, we conceptualize the player in possession of the ball and an immediate defender from an ecological perspective and discuss the implications for future research and applied practice. From a scientific-based perspective, the adoption of an ecological approach to perception and action affords the use of a ball-centric framework, and so allows for the unity of coaching and scientific domains. This ball-centric approach cannot be superficially imposed on any theoretical framework. Conversely, it requires a necessary ontological commitment that allows the specification of a conceptualization (e.g., of the game), a description of the concepts and relationships that emerge and exist for an agent (-player) or a group of agents (-teams), so that the framework aids understanding of the fundamental principles of the game. This new ontology, based on Gibson’s^28^ discipline of ecological psychology, and work that builds off his seminal arguments^22,29^, rejects the organism-environment dualism and eschews explanations that treat these in isolation from each other. Instead, an ecological approach treats the organism-environment as a coupled-dynamic system subject throughout to the same kind of laws, called laws of ecological physics^23^.

The ball-centric approach has been adopted to study how the displacements of the ball via passes change the local informational gradient and collective dynamics of players in a passing drill^30^. In this article, the goal is to tackle a second form of in-game ball dynamics, dribbling, and the player-ball system that emerges during dribbling actions. Under the assumption that during dribbling activities, the player dribbling the ball changes other players’ informational gradients, and so the activities of the whole system, we aim to develop a mathematical model to quantify the dynamics that emerge from this player-ball system.

Previous literature on the skill of dribbling has determined that it is one of the most common technical actions that take place in a football match^31^ and a determining factor differentiating the most talented players^32^ and in the development of young players^33^. Previous research on dribbling also relates to skill-acquisition; biomechanics^34^; and the physiological implications of dribbling^35,36^. However, these papers do not seek to explore the in-game dynamics of the player-ball system and its changes in a systematic way, building from a theory that illuminates how the system (game) works.

In this paper, we present a ball-centric mathematical model that captures the trajectories of 1v1 dribble segments, while preserving ecological-based principals. A 1v1 dribble scenario or segment is defined as a continuous event where a single attacker attempts to dribble a single defender, which lasts until the attacker has lost possession of the ball or the defender has been overtaken. For the sake of brevity, we hereafter use dribble to refer to a 1v1 dribble segment. The proposed model for dribbling is inspired by a mathematical model introduced by Keller for short-distance competitive running^37,38^, as well as the steering dynamics model from Fajen and Warren^39^, whereby we treat the ball and the dribbler as a coupled dynamical system. The model consists of three key ingredients: (1) the attacker behavior is goal-directed, and depends on the success of dribbling the ball towards desired spots along an intended path; (2) the defender acts in relation to the active movement of the attacker, which in turn acts in relation to the defender activity, hence both players’ motion is mutually influenced; (3) in-game real tracking data of high-level professional football players is used to fit the model parameters, thus enabling the quantification of dribbling skills beyond traditional metrics such as dribble success rate. Hence, the proposed framework overcomes prior efforts to capture the dynamics of dribbling^40^, in which a two-stage approach of kicking the ball and pursuing the kicked ball of an attacker dribbling the ball around a static defender to a goal was proposed.

This paper is organized as follows: in “Model development” section, we develop a mathematical model that quantifies the system generated by a dribbler and their immediate defender, using parameters characterizing both physical and behavioral aspects of footballers; in Section “Model Validation” we demonstrate the effectiveness of the model in predicting trajectories of elite football players in real dribbling scenarios, using data provided by the San Jose Earthquakes in the Major League Soccer (MLS); in “Analysis of parameter distributions” section we use the model parameter values that arise from fitting the model to real tracking data to conduct several statistical analyses depending on different game conditions; and finally in Section “Quantifying the Impact of Parameter Variations on Dribble Quality” we investigate how changes in the model parameters impact the quality and outcome of dribbles.

## Results and discussions

### Model development

Our starting point is the original one-dimensional Keller model^37^ for competitive running

1 $$\begin{aligned} {\dot{v}}(t) = -\dfrac{1}{\tau }v(t) + f(t), \end{aligned}$$

where *v*(*t*) is the runner velocity and the dot indicates a derivative with respect to time, *f*(*t*) is the positive force per unit mass exerted by the runner, assumed to be constant throughout the race, and $\tau >0$ is a constant modeling the physiological resistances felt by the runner.

**Figure 1:**
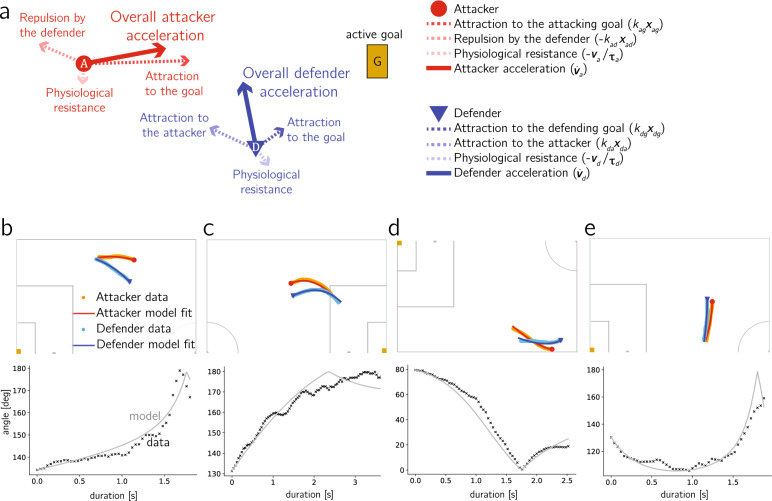
Attacker-defender dribble model. (**a**) Schematic illustration of the vector components that constitute the model. (**b**–**e**) Comparison between tracking data and model results for four dribble events. Top: Player trajectories. Bottom: Goal-defender-attacker angle as a function of dribble duration. Legend is shared across subfigures.

The Keller model has proven to be successful in modeling straight-line running behavior^37^. However, in order to adapt it to soccer, the model must be expanded to allow for 2D forces and trajectories. To introduce directionality, we characterize dribbling situations based on three main agents: the player in control of the ball, referred to as *attacker* and represented by subscript *a*; the goal that this player is attacking, referred to as *active goal* and represented by subscript *g*; and the opposing player who is closest to the attacker, referred to as *defender* and represented by subscript *d*. To capture the two players’ trajectories with the model, we rewrite (1) as a set of two 2D ordinary differential equations—one for the attacker and one for the defender—and choose $\varvec{f}(t)$ in terms of the attractive/repulsive interactions between the agents, as sketched in Fig. 1a. For the attacker, we postulate that the force per unit mass is dictated by a constant attraction to the active goal and a constant repulsion from the defender $\varvec{f}_a(t) = k_{ag}\varvec{x}_{ag}(t) - k_{ad}\varvec{x}_{ad}(t)$, whereas the defender is modeled by a constant attraction to their own goal in order to stay between the goal and the attacker and the constant attraction to the attacker to intercept the ball $\varvec{f}_d(t) = k_{dg}\varvec{x}_{dg}(t) + k_{da}\varvec{x}_{da}(t)$. In these expressions, $\varvec{x}_{ij}(t)$ represents the unit vector from agent *i* to agent *j* at time *t*, and $k_{ij}$ is the nonzero attraction/repulsion constant coefficient between agents *i* and *j*. Thus, unit vector $\varvec{x}_{ij}(t)$ and constant coefficient $k_{ij}$ respectively determine the direction and the magnitude of corresponding interactions between the agents.

In real game situations, we expect the magnitude of those interactions to depend on the distance between the agents, especially between the attacker and the defender (e.g. attacker and defender may not feel the attraction if they are far away from each other). To account for this distance dependence in a simple way, we assume that $k_{ij}$’s are zero when the attacker and the defender are far away and nonzero constants when the attacker and the defender are close to each other—in this work, we set a conservative criteria of attacker-defender distance below 10 meters and only focus on those dribbles in the downstream analysis, see also Fig. S1b and Section S1.5. We acknowledge that more complex models can be built to capture the delicate aspects of different interactions between the agents, but in this work, we focus on introducing the simplest model possible and demonstrating that already enables the quantification of dribbling skills.

Consequently, the Keller-inspired model for the attacker and defender can be written as follows:

2a $$\begin{aligned} \dot{\varvec{v}}_a(t)&= -\dfrac{1}{\tau _a}\varvec{v}_a(t) + k_{ag} \varvec{x}_{ag}(t) - k_{ad}\varvec{x}_{ad}(t)\,, \end{aligned}$$

2b $$\begin{aligned} \dot{\varvec{v}}_d(t)&= -\dfrac{1}{\tau _d}\varvec{v}_d(t) + k_{dg} \varvec{x}_{dg}(t) + k_{da}\varvec{x}_{da}(t)\,. \end{aligned}$$

It must be noted that the force terms $\varvec{f}(t)$ are related to the players’ athletic capabilities and therefore should have physically-reasonable magnitudes. The equilibrium analysis for this system may be found in Section S2 of the SI. In order to more easily apply this physiological constraint to the magnitude of the $\varvec{f}(t)$ components, we rearrange Eq. (2) and introduce the parameters $\beta _a = k_{ag}/k_{ad}$ and $\beta _d = k_{dg}/k_{da}$, as well as $\alpha _a = \dfrac{k_{ad}}{\left| k_{ad}\right| }\tau _a \left\| \varvec{f}_a(t)\right\|$ and $\alpha _d = \dfrac{k_{da}}{\left| k_{da}\right| }\tau _d \left\| \varvec{f}_d(t)\right\|$. Hence, the dribbling model that we propose is given by the following set of equations:

3a $$\begin{aligned} \dot{\varvec{v}}_a(t)&= -\dfrac{1}{\tau _a}\varvec{v}_a(t) + \dfrac{\alpha _a}{\tau _a} \dfrac{\beta _a \varvec{x}_{ag}(t) - \varvec{x}_{ad}(t)}{\left\| \beta _a \varvec{x}_{ag}(t) - \varvec{x}_{ad}(t)\right\| }\,, \end{aligned}$$

3b $$\begin{aligned} \dot{\varvec{v}}_d(t)&= -\dfrac{1}{\tau _d}\varvec{v}_d(t) + \dfrac{\alpha _d}{\tau _d} \dfrac{\beta _d \varvec{x}_{dg}(t) + \varvec{x}_{da}(t)}{\left\| \beta _d \varvec{x}_{dg}(t) + \varvec{x}_{da}(t)\right\| }\,, \end{aligned}$$

which is hereafter referred to as attacker-defender model. The $\beta$ parameters model the behavior of each player in relation to the other agents: $\beta _a$ represents the attacker’s *aggressiveness*, computed as the ratio between the attraction to the active goal and the attraction to the defender; $\beta _d$ represents the defender’s *hesitance*, computed as the ratio between attraction to the active goal and attraction to the attacker. The remaining parameters have a physiological interpretation stemming from Keller’s original work^37^, whereby $\alpha$ represents the top speed attainable by the player in a specific dribble situation (and can thus vary with fatigue, player’s level of effort, player/game conditions, etc.), $\tau$ is a decay constant measuring the inner resistances felt by the player (in seconds) and $\alpha /\tau$ is the force per unit mass exerted by the player in the given dribbling situation, see Keller’s work^37^ for further details. Since the underlying attraction/repulsion constants *k* may be positive or negative, the behavioral parameters $\beta$ and top velocity parameters $\alpha$ are not constrained to be positive. The assumed player behavior (attacker attracted to goal and repelled from defender, defender attracted to goal and attacker) only occurs when $(\beta _a,\beta _d,\alpha _a,\alpha _d)>0$, whereas other less frequent situations arise whenever one or more of these parameters are negative, which we briefly discuss in Section S8 of the Supplementary Information (SI). In this article, we choose a top player velocity constraint of $\left| \alpha \right| < 10.2$ m/s based on the maximum value of player speed obtained from our dataset; $\tau$ is a positive parameter with a reasonable lower bound of 0.9 s, which is the value previously reported by fitting the model to short-distance track records based on Keller’s report^37^; the behavioral $\beta$ parameters are a ratio, which given the lack of existing data, for now we take as unconstrained.

To evaluate the player’s trajectories, we need the initial location of all three agents and the initial velocities of both players, in addition to a feasible configuration of parameter values. Then, by numerically solving Eq. (3) we retrieve the model-predicted player trajectories. Alternatively, we can leverage player trajectory data from real in-game situations and use the proposed model to extract values for the parameters $\beta$, $\alpha$ and $\tau$, for which the discrepancy between the actual and predicted player trajectories is minimized.

### Model validation

To validate that the model accurately captures dribble behavior, we test whether the trajectories predicted by the model are similar to those from real in-game player trajectories. The San Jose Earthquakes in the Major League Soccer (MLS) provided us with player tracking data (sampled at 25Hz), as well as discrete annotations (attacker, defender and timestamp) on dribbling situations for 17 home games of the 2019 season, both collected by Second Spectrum (SS). In order to convert the discrete 1v1 annotations to dribble segments, starting from the annotated timestamp we assume the dribble segment starts when the attacker is in control of the ball ($<0.5m$), and ends when either the attacker is no longer in possession of the ball ($>1m$) or the attacker is far from the defender ($>10m$), see Section S1.1 in the SI for further details.

We assess the ability of the model to reproduce real dribble trajectories by quantifying the fitting error for each dribble, computed by comparing the trajectories from the model and the actual player trajectories from the tracking data. An optimization process is performed for each dribble instance in order to extract the set of six parameters that minimize the fitting error, defined in Eq. (4) in Methods. We find that 80% and 86% of dribbles exhibit an error below 0.1 in the attacker’s and defender’s trajectory, respectively. In addition, a total of 73% and 43% dribbles have errors for both players below 0.1 and 0.05, respectively. For reference, an error of 0.1 represents an average of 10% frame-by-frame deviation from the actual data along the entire duration of the dribble. We can therefore argue that, despite the low dimensionality of the model proposed above, it is able to effectively capture the dynamics of the participating players, and enables the parameterization of these scenarios using only three parameters per player (or per trajectory): two parameters to describe physical condition (top speed attainable and inner resistances felt by player) and one behavioral parameter that measures either attacker aggressiveness or defender hesitance. Examples of accurately fitted pairs of trajectories, with both errors below 0.02 (less than 2% frame-by-frame deviation) are shown in Fig. 1b–e, and we refer the reader to Section S4 and Fig. S7 in the SI, for six additional examples of dribble segments where the computed model trajectories match the tracking data within 1%.

It should be kept in mind that our model assumes 1v1 situations and simplifies the dribble dynamics. Thus, there exist several cases where our model exhibits limited accuracy, owing to e.g.: sudden change in trajectories of the players (since our model assumes smooth trajectories); the presence of multiple defenders (we consider only the closest defender labeled by SS); or the influence of pitch bounds (the model assumes the 1v1 occurs on an unbounded domain). Some examples of trajectories with both errors above 0.1, that correspond to the aforementioned limitations, are discussed in Section S8 of the SI, along with potential avenues to circumvent those.

### Analysis of parameter distributions

The process of fitting the dribble trajectories with the proposed model gives rise to six parameter values per dribble, which along with the initial positions and velocity of the players, fully characterize the dribble. The empirical distributions of these six parameters have been further analyzed by slicing the dribbling dataset along different football-specific attributes, in order to determine whether statistically significant differences arise for different values of the attributes. The analysis is focused on the behavioral parameters $\beta _a,\,\beta _d$ as well as the parameters modeling the force per unit mass exerted by the players $\alpha _a/\tau _a,\; \alpha _d/\tau _d$; the attributes considered are the positional role of attacker and defender (forward, midfielder, back), whether the attacker is a top-10 MLS dribbler^42^ and the pitch location where the dribble started.

The dribbles considered for this study are further filtered by error, parameter positivity and sensitivity. Lower fitting errors indicate a more accurate model representation of the dribble dynamics; positive parameter values confirm that the interaction factors that we assume in the model (attacker attracted to active goal and repelled from defender, defender attracted to active goal and attacker) primarily govern the trajectory of the players; sensitivity quantifies how much the predicted player trajectories change when player parameters are modified, hence high parameter-sensitivity ensures that the parameter value well-represents that specific dribbling situation. A more detailed explanation of these filtering criteria can be found in the "Methods" section, as well as Sections S1.4 and S3 of the SI.

**Figure 2:**
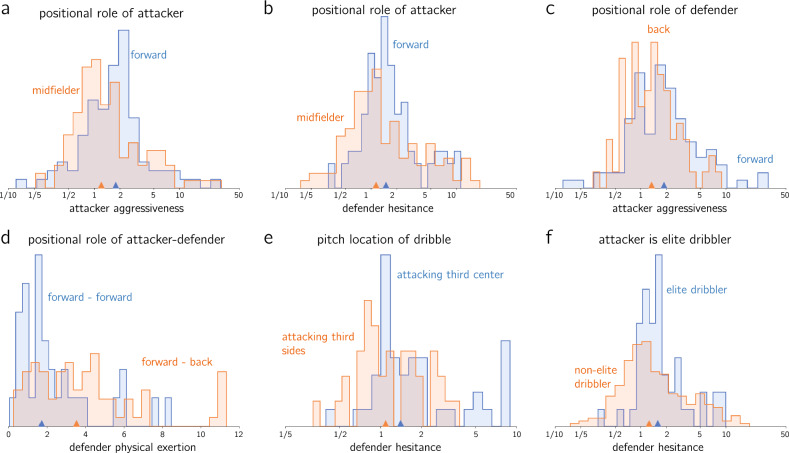
Normalized histograms of empirical distributions for selected parameters and categories, with the median of each distribution shown as upward triangle. Attacker/defender refers to whether the player is in possession of the ball when dribbling, whereas back/midfielder/forward refers to the player positional role within the team’s lineup: (**a**) attacker aggressiveness $\beta _a$ comparing when the player in possession is either a forward or a midfielder; (**b**) defender hesitance $\beta _d$ comparing when the player in possession is either a forward or a midfielder; (**c**) attacker aggressiveness $\beta _a$ when the player not in possession is either a forward or a back ; (**d**) defender physical exertion $\alpha _d/\tau _d$ comparing dribbles executed by forwards when the player not in possession is either a back player or another forward; (**e**) defender hesitance $\beta _d$ comparing when the dribble started from either the flanks or the center of the attacking third; (**f**) defender hesitance $\beta _d$ slicing the player in possession between elite and non-elite dribblers.

For each parameter, we categorize all filtered dribbles according to one of the above attributes (player positional role, elite dribbler and dribble location), and then perform all possible pairwise comparisons of the parameter empirical distribution using a Kolmogorov–Smirnov (KS) two-sided test. The null hypothesis of the test is that both empirical distributions are drawn from the same underlying distribution, hence if the *p*-value is lower than *p* we may reject the null hypothesis with a significance of $1-p$. In order to ensure the analysis is statistically valid, a minimum of 25 dribbles per category is prescribed to apply the KS test. For each parameter, we report the amount of pairwise categories where the null hypothesis may be rejected at 1% and 5% significance. In the following subsections, we explore in detail six examples of parameter distributions that exhibit significant differences for distinct values of the attributes. The observed differences between attributes for these examples are consistent with football intuition, see Fig. 2.

#### Player positional role

The goal of this analysis is to quantify potential differences in parameter distributions between different player types, based on their natural/preferred playing position. We divide the players in three groups (back/midfielder/forward) based on their position in the lineup^43^. We use this nomenclature to distinguish the positional role of a player within the team’s lineup from whether the player is in possession of the ball (attacker) or not in possession of the ball (defender) during the dribble.

We first look at the positional role of the attacker only (3 categories, $$\left( {\begin{array}{c}3\\ 2\end{array}}\right)$$ = 3 possible comparisons), then only the positional role of the defender (3 categories, 3 possible comparisons), and finally the combined positional role of attacker and defender (9 categories, 36 possible comparisons). Out of the total 42 comparisons, the percentage of pairwise comparisons where the null hypothesis can be rejected at 5% significance is 19% for $\beta _a$, 5% for $\beta _d$, 2% for $\alpha _a/\tau _a$ and 14% for $\alpha _d/\tau _d$.

Fig. 2a–d shows four statistically significant comparisons, which we select based on their football-specific interpretation. Focusing only on the positional role of the attacker, in Fig. 2a we show the empirical distributions of attacker aggressiveness $\beta _a$ for forwards vs midfielders when attacking (*p*-value $5\mathrm{e-}3$). We see that forwards exhibit 50% higher aggressiveness values (in median) than midfielders when handling the ball in the dribble, and are therefore less mindful of the opponent. This agrees with forwards’ primary task of getting past the defender to score, which could lead to an increased willingness to take the potential risk of losing the ball. Furthermore, forward players generally dribble closer to the opposing goal, reducing the risk of a dangerous situation in which they lose the ball. Next, in Fig 2b, we analyze the distributions of the defender hesitance $\,\beta _d$ when the player handling the ball is either a forward or a midfielder (*p*-value $1.8\mathrm{e-}2$). We see that when defenders are facing a forward, they are 40% more hesitant (in median) than when facing a midfielder, which is explained by the generally higher dribbling skill level of forward players compared to midfielders, posing more danger to the defenders. In Fig. 2c, we slice the attacker aggressiveness data $\beta _a$ based on the positional role of the defender. We observe that the attacker exhibits 46% higher aggressiveness towards the active goal (in median) when facing a forward than when facing a back player (*p*-value $8.2\mathrm{e-}3$), implying a lower perceived defensive skill level of forwards compared to back players. Finally, in Fig. 2d, we compare the distributions of the physical exertion parameter $\alpha _d/\tau _d$ for a forward–forward dribble vs a forward–back dribble, where the first player represents the attacker and the second the defender (*p*-value of $3.1\mathrm{e-}3$). The data shows that when facing a forward player in a dribble, the defender exhibits 50% lower levels of physical exertion (in median) if they are themselves a forward compared to them being a back player, which is explained by the perceived lower defensive skill level of forwards compared to back players.

#### Dribble start location

Next, we analyze differences in parameter values based on the start location of the dribble. We divide the pitch into three zones along its length: attacking, central, and defending (after proper normalization to ensure a unique attacking direction), and two zones along its width: side and central, hence we are left with 6 categories (15 pairwise comparisons). The percentage of pairwise comparisons where the null hypothesis can be rejected at $\ 5\%$ significance is $\ 7\%$ for $\beta _a,\,\beta _d$, $\ 0\%$ for $\alpha _a$ and $\ 27\%$ for $\alpha _d$. Amongst the pairwise comparisons that exhibit statistical significance, we depict in Fig. 2e the one with the lowest *p*-value ($2.1\mathrm{e-}2$), namely the distributions of defender hesitance for dribbles starting in either the flanks or the center of the attacking third. For the latter case, the defender exhibits $\ 27\%$ more hesitance (in median), showing that defenders are less aggressive and more mindful of their goal when defending a dribble that starts in the center of the attacking third compared to the flanks of the attacking third, where the downside of being surpassed by the attacker is inferior.

#### Top-10 dribblers

Finally, we aim to quantify differences in parameter distributions between players classified as top-10 dribblers of the MLS and other players. In order to make this classification, we use the list of top-10 MLS dribblers in 2019 and 2020 according to the MLS website^42^. In the dataset considered, 21.5% of dribbles featured one of these elite MLS dribblers as the attacker.

Since there are only two categories (top-10 dribbler or not), there is only one possible pairwise comparison per parameter. We find that the null hypothesis can only be rejected for $\beta _d$, with a *p*-value of $5.6\mathrm{e-}3$. The distribution of $\beta _d$ for each of the two categories is shown in Fig. 2f. Defenders facing an elite dribbler exhibit 27% more hesitance than when facing a non-elite dribbler (in median). Indeed, defenders are more reluctant of being overtaken by the highly-skilled opponent if actively going towards them, choosing to be more mindful of their own goal instead.

### Quantifying the impact of parameter variations on dribble quality

The next step after analyzing the distribution of parameters coming from the model optimization is to quantify how dribble trajectories change when the model parameters vary. This allows for predicting changes in dribble quality based on potential changes in either behavior ($\beta _a,\,\beta _d$) or improvement of physical condition ($\alpha _a,\,\alpha _d$), and could thus help identify and compare options for player development and developing game plans.

**Figure 3:**
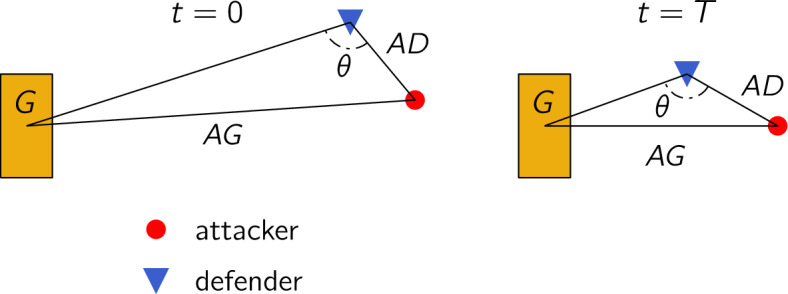
Representation of the start ($t=0$) and end ($t=T$) of a dribble segment with attacker, defender and active goal, highlighting the relevant quantities required define the score: attacker-defender-goal angle $\theta$, attacker-goal distance *AG* and attacker-defender distance *AD*.

In order to be able to compare different output trajectories from the model, we first define a simple metric, hereafter referred to as dribble score, that allows us to quantitatively rate dribbles. We define the dribble score $z \in [0,1]$ as the weighted sum of three terms: the change in angle of the attacker-defender-active goal from start ($t=0$) to end ($t=T$) of dribble (change in positional advantage as a result of the dribble), the relative distance traveled to the goal by the attacker (how closer to the goal the attacker could advance by dribbling), and the final distance between attacker and defender (how effectively the attacker avoided the defender and reliably maintained the possession of the ball). A visual representation of the score is shown in Fig. 3, and we also refer the reader to “Dribble Score” section in Methods for extensive details on how the score is evaluated for individual dribble segments. Although we demonstrate our analysis with this relatively simple score metric, we acknowledge that this is not the only way to evaluate dribbles and more complex dribble scores (e.g. different weights for those three elements, incorporating temporal aspects of the elements such as ${\dot{\theta }}$) can be introduced depending on the objective of the analysis.

**Figure 4:**
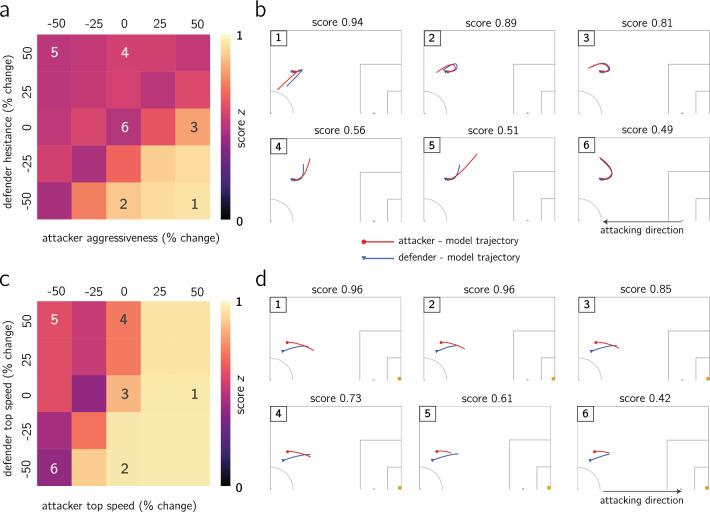
Impact of behavioral parameter variation (**a**,**b**) on dribble 1 and top speed variation (**c**,**d**) on dribble 2: (**a**) heatmap of predicted scores for simultaneous $\pm 25,\pm 50\%$ variations in attacker aggressiveness $\beta _a$ and defender hesitance $\beta _d$ (heatmap generated using seaborn library^44^ in python 3.7); (**b**) simulated trajectories and predicted score for prescribed percent change in behavioral parameters; (**c**) heatmap of predicted scores for simultaneous $\pm 25,\pm 50\%$ variations in attacker (resp. defender) top speed $\alpha _a$ (resp. $\alpha _d$) (heatmap generated using seaborn library^44^ in python 3.7); (**d**) simulated trajectories and predicted score for prescribed percent change in top speed parameters.

The analysis in this section is split into two parts. First, we demonstrate how combining the attacker-defender model with the dribble score can be useful to examine the impact of parameter changes, by focusing on two individual exemplary dribbles. For each of the two dribble segments, we re-run the model (3) with the same initial conditions while prescribing parameter variations (up to $\pm 50\%$), though ensuring the parameter constraints are not violated, and for each variation we obtain a new pair of attacker-defender trajectories. We depict the impact of the parameter variations as a heatmap, where each cell is colored according to the dribble score achieved with the corresponding parameter variations (the center cell corresponds to the dribble score without parameter variations), see Fig. 4a,c. Moreover, for each dribble we examine the effect of five parameter variations in the actual player trajectories, in addition to the baseline trajectories without modifications, see Fig. 4b,d sorted in descending score. For the first dribble, as in Fig. 4a,b, we quantify the effects of variations in the behavioral parameters of both the attacker and the defender ($\beta _a,\,\beta _d$), and we see that the baseline parameter configuration is the one rendering the lowest scores. For the second dribble, see Fig. 4c,d, we explore changes in the top speed parameters $\alpha _a,\,\alpha _d$ for both players. Additional examples of scores and predicted trajectories when modifying the parameters for other dribbles may be found in Sections S6 and S7 of the SI.

**Figure 5:**
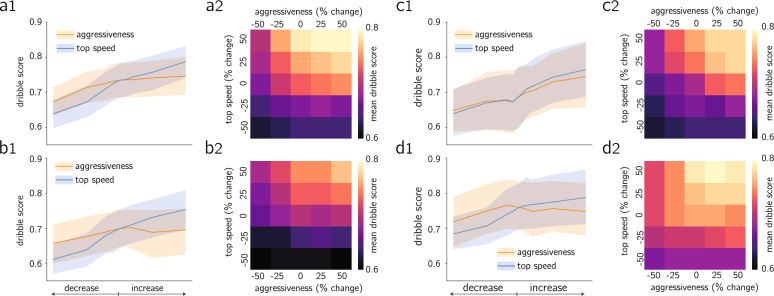
Is it more beneficial to improve top speed and/or aggressiveness when dribbling? (a1,b1,c1,d1) Predicted mean score with 95% confidence interval for four different players when changing $\beta _a,\,\alpha _a$ independently up to $\pm 50\%$; (a2,b2,c2,d2) predicted mean score for four different players when changing $\beta _a$ and $\alpha _a$ simultaneously. Results suggest all players benefit from increase in top speed, which is more advantageous than the same amount of increase in aggressiveness; players (**a**,**c**) exhibit an increase in mean score when increasing aggressiveness, although much more pronounced for player (**c**); player (**b**) is operating at optimal aggressiveness; player (**d**) should decrease their aggressiveness to improve their scores.

After looking at the individual dribble level, we shift the focus to aggregate score metrics across players. This allows us to evaluate the average impacts of changing the player’s behavior and physical condition. In this analysis, we only focus on quantifying the impacts of variations of the attacker, namely $\alpha _a$ and/or $\beta _a$, although changes on the parameters of the defender may be analyzed analogously. To that end, we choose four different players in our dataset for who we observe distinct impact of the parameter variations. The mean dribble score values for these four different players under parameter variations is shown in Fig. 5. More specifically, Fig. 5a1,b1,c1,d1) show the impact of changing either $\alpha _a$ or $\beta _a$ on the mean dribble score, along with the 95% confidence interval, whereas Fig. 5a2,b2,c2,d2 show the impact of simultaneous changes in both $\alpha _a,\,\beta _a$ as a mean score heatmap. The remaining players in our dataset (for which we have at least 10 dribbles) are analyzed in Fig. S13 in the SI.

For all four players, increasing top speed $\alpha$ translates into higher scores, e.g. a 25% increase in top speed leads to a 8% increase in dribble score for player (c), 5% for player (b), 3% for player (a) and 2% for player (d). This is in agreement with the football intuition that the ability to run faster increases the likelihood of beating a defender in 1v1 interactions. Similarly, a decrease in speed is followed by a decrease in scores, for instance a 25% decrease in top speed results into an 8% reduction in dribble score for players (a,b), 4% for player (b), 7% for player (d) and 3% for player (c). Conversely, the effects of changing $\beta _a$ attacker aggressivenesses are different across the four players. For players (a) and (c), a 50% increase in $\beta _a$ leads to increases of 2% and 8% in the dribble score, respectively. However, for players (b) and (d), a 50% increase in $\beta _a$ causes a decrease of 1% and 2% in the dribble score, respectively. From Fig. 5b1,d1, we see that player b seems to be operating at an optimum $\beta _a$ whereas player (d) is operating beyond its optimal $\beta _a$ value. Finally, player (a) experiences a 9% reduction in dribble score when for a 50% decrease in aggressiveness, compared to a 6% for the other players.

Moreover, we also show the combined effects of changing $\beta _a-\alpha _a$ as heatmaps in Fig. 5a2,b2,c2,d2. For instance, a decrease of 50% in top speed for all players negates further effect on the scores from changes in aggressiveness. The analogous observation may be made for a 50% reduction in aggressiveness, whereby top speed changes become far less relevant. Hence, when inspecting how the dribbling skills of a given player may be enhanced, it is paramount to account for both the player aggressiveness and top speed simultaneously.

We believe that these analyses demonstrate the possibility of using the proposed attacker-defender model to quantify player attributes that have not been quantifiable before. These differences in score sensitivity to $\beta _a$ suggest potential opportunities for personalized player development and scouting. An example of the former would be that for player (a) it is beneficial to work on both taking more risks and increasing top speed, while for player (d) it is more beneficial to improve their top speed and reduce their aggressiveness in taking risks. Regarding player scouting, this methodology can be used to assess player potential beyond traditional statistics (such as dribbles attempted or completed) and establish a baseline to compare players.

### Conclusion

We have presented a mathematical attacker-defender model to capture the dynamics of 1v1 dribbling in football. The model consists of a system of two ordinary differential equations, which can be solved to obtain attacker and defender’s trajectories. Each equation is described by a behavioral parameter, measuring either attacker’s aggressiveness towards the goal or defender’s hesitance in chasing the attacker, and two physical parameters measuring the top speed and the inner resistances felt by the player. We use real in-game tracking data from elite MLS footballers to fit the parameters of the attacker-defender model, showcasing the model’s ability to accurately capture the player trajectories while dribbling. Additionally, this fitting process with real data enables the extraction of the aforementioned player-specific dribbling parameters. The parameter distribution analysis enables us to ascertain that there are statistically significant differences between the parameters depending on the positional role of the player, the dribble location or dribbling record of the player, and hence that these parameters constitute novel metrics to characterize the dribbling skills of professional football players, which can potentially be used in player development and scouting. By analyzing the impact of parameter changes on the player’s trajectories we are able to identify player-specific potential training and performance strategies. Moreover, the framework presented here can be used to construct game plans such as which defender to target, which attacker should be the dribbling focal point, where on the pitch should the dribble start, etc. and provide a quantitative metric to assist scouting by assessing the dribbling skills of any player in terms of measurable behavioral and physical parameters.

In terms of future work, two categories may be identified: (1) model improvement requires extending the model beyond 1v1 dribbles by including additional equations/terms for other players and the sidelines, see Section S8. Furthermore, the model equations could be extended to include a skill-related parameter, which would allow for extending the model beyond physical and behavioral parameters; (2) player-player and player-positional role analyses that are specific to an individual player, with the objective of tailoring line-ups or in-game strategies to certain opponents or game situations. The current limitation is the lack of sufficient dribble data to perform these studies.

## Methods

### Dataset

For this study, we have used data provided by the San Jose (SJ) Earthquakes collected by Second Spectrum (SS). The data, which comprises 17 games SJ played at home in the 2019 season, consists for each game of ball and (*x*, *y*)-player position data sampled at 25Hz (tracking data), as well as a log of all 1v1 situations that occurred indexed by the minute and second (cents of second not available), specifying the attacking and defending players (event data). The pre-processing step is detailed in Section S1.1, resulting in a dataset of 2714 dribbles, where 1691 of those are executed by SJ players.

We have received permission from San Jose Earthquakes, who owns the player tracking data provided by Second Spectrum, to use the data for this research. Second Spectrum was authorized to collect the data under a partnership with San Jose Earthquakes. Informed consent was obtained from all subjects. All methods were carried out in accordance with relevant guidelines and regulations, and the authors received human research ethics approval to conduct this work from the Committee on the Use of Humans as Experimental Subjects (COUHES-MIT).

### Parameter fitting

In order to fit the proposed model to a given dribble, an optimization step is performed to compute a set of parameters that minimize the combined error of attacker and defender trajectories, (see Section S1.2). An error metric is introduced to quantitatively assess how accurately the model is able to predict the trajectories of both players during the dribbling event. The distance-normalized error metric for an arbitrary dribble is defined as

4 $$\begin{aligned} \varepsilon _{p} = \dfrac{\sum _{t=0}^{T}\left\| \textbf{r}_p(t) - \widetilde{\textbf{r}}_p(t)\right\| }{\sum _{t=1}^{T}\left\| \textbf{r}_p(t) - \textbf{r}_p(t-1)\right\| }\dfrac{1}{T} \end{aligned}$$

where $p \in \lbrace a,\,d \rbrace$ refers to either the attacker or the defender, *T* is the dribble duration in data frames, $\textbf{r}_p(t)$ (resp. $\widetilde{\textbf{r}}_p(t)$) is the (*x*, *y*) tracking data (resp. model trajectory) position of player *p* at instant *t*. The physical meaning of this error metric is the average fractional deviation of player position at each frame (average positional deviation divided by player travel distance). An exploratory analysis of dribble duration and distance after pre-processing, as well as fitting errors, for the entire dataset may be found in Section S1.3 and Fig. S1(a) of the SI.

### Dribble filtering

Since one of the objectives of this work is to extract meaningful physical and decision-making insights from players regarding dribbling situations, we need to carefully select the dribbles that will be analyzed. To that end, we filter the dribbles based on the following criteria: minimum distance of 2 m traveled, model error below 0.1 for both players and parameter positivity (reduced the dataset to 1573, 1153 and 623 dribbles in turn). Further details on those filtering criteria can be found in Sections S1.4 and S3. The 623 filtered dribbles are uniformly distributed with respect to game time and first/second half of the match (53%/47%). The proportion of dribbles carried out in the opposing half of the pitch was slightly higher than in the own half of the pitch (60%/40%). The home players were the ball handlers in 64% of the filtered dribbles, while 60% of the dribbles were performed by the team that ended up winning the match.

#### Parameter sensitivity

We need to consider only dribbles where the quality of the model fit is directly impacted by the value of the parameters. In other words, for each parameter under consideration we filter out dribbles where significant variations on the value of that parameter did not translate into significant variations in the model error.

Sensitivity study is performed by perturbing one of the parameters (say $\beta _a$) by a given percent value $\pm \rho$ while leaving the others fixed, then simulating the new attacker and defender trajectory using these new set of parameters with our model, and evaluating the new error. We then deem this dribble to be sensitive with respect to $\beta _a$ if the relative change in error is at least $\left| \rho \right|$. The results derived in “Analysis of parameter distributions” section are evaluated with $\rho =\pm 25\%$, for a grand total of (293, 274, 535, 522) dribbles for $(\beta _a,\,\beta _d,\,\alpha _a/\tau _a,\, \alpha _d/\tau _d)$ (since for each parameter analysis we only use dribbles that are sensitive to that parameter). Similarly, the results in Section “Quantifying the Impact of Parameter Variations on Dribble Quality” are evaluated with dribbles that are sensitive to at least one of the $\rho =\pm 10\%,\,\pm 25\%,\,\pm 50\%$, leaving us with (377, 359, 541, 531) dribbles for $(\beta _a,\,\beta _d,\,\alpha _a,\, \alpha _d)$.

### Dribble score

To evaluate dribbles, we propose the following scoring metric. The score *z* is defined as

5 $$\begin{aligned} z = \dfrac{1}{3}(z_{\theta } + z_{AG} + z_{AD}), \end{aligned}$$

and the three terms are individually defined between 0 and 1, hence $z\in [0,1]$, involving the angle, the attacker-goal distance and the attacker-defender distance, see Fig. 3. The first term $z_\theta$ accounts for the change in attacker-defender-goal angle $\theta \in [0,\pi ]$, namely $\Delta _\theta = \theta _{t=0} - \theta _{t=T}$ (where $\theta = 0$ corresponds to dribbles with the attacker in front of the defender and both aligned with the goal and $\theta = \pi$ to the attacker behind the defender), and represents how well the attacker can see the route to the goal without the interruption of the defender in between. The second term $z_{AG}$ accounts for the relative change in attacker’s distance to the goal (referred to as AG) and may be evaluated as the empirical cumulative distribution function (ECDF) of the variable $\Delta _{AG} = (AG_{t=0}-AG_{t=T})/AG_{t=0}$, thus higher scores are awarded to dribbles where the attacker gets closer to the goal. Since the construction of the scoring system is independent of the dribble fitting, the dribbles considered to construct the ECDFs (for $\Delta _\theta$ and $\Delta _{AG}$) have only been filtered by traveled distance (1573 dribbles). Once the ECDFs have been computed, they are queried for each individual dribble’s $\Delta _\theta$ (resp. $\Delta _{AG}$) to obtain $z_\theta$ (resp. $z_{AG}$).

The third term $z_{AD}$ accounts for the distance between the attacker and defender at the end of the dribble $AD_{t=T}$. For this term, data-driven approach is not optimal since the final attacker-defender distance has already been used as a pre-processing criterion. So instead, we leverage football knowledge that high scores should be awarded to dribbles finishing far from the defender, while the pressure felt by the attacker from the defender is generally proportional to the distance between them only when the defender is close by, and model it with using a piecewise linear unit with a 2 m cutoff, whereby dribbles where the distance is greater than 2 m the score $z_{AD}$ is 1 and between 0 and 2 m the score $z_{AD}$ is linearly interpolated between 0 and 1. Further details and analyses on this score, as well as other possible scoring criteria, are extensively discussed in Section S5 of the SI.

### Supplementary Information

Supplementary Information.

## Data Availability

The data used for this study was collected by the San Jose Earthquakes in the MLS 2019 season. Due to media and data rights, the datasets are not publicly available, but can be requested from the corresponding author together with a viable research proposal and with permission of the San Jose Earthquakes.
