# Supplementary material for: Measuring skill via player dynamics in football dribbling
Source: Sci Rep. 2023 Nov 3;13:19004. doi: 10.1038/s41598-023-45914-6 (PMC10624867; doi:10.1038/s41598-023-45914-6)
Supplement: Supplementary file 1 — Supplementary Information. [file 41598_2023_45914_MOESM1_ESM.pdf]

Supporting Information:

Measuring skill via player dynamics in football dribbling

Lukas Brink[1,†], Seung Kyun Ha[2,†,*], Jack Snowdon[3,†], Ferran Vidal-Codina[1,†], Bobby Rauch[3,‡], Fan Wang[4,‡], David Wu[5,‡], Maurici López-Felip[6,7], Christophe Clanet[8], and Anette E. Hosoi[4]

[1]*Department of Aeronautics and Astronautics, Massachusetts Institute of Technology, Cambridge, MA 02139, USA*
[2]*Department of Chemical Engineering, Massachusetts Institute of Technology, Cambridge, MA 02139, USA*
[3]*Department of Electrical Engineering and Computer Science, Massachusetts Institute of Technology, Cambridge, MA 02139, USA*
[4]*Department of Mechanical Engineering, Massachusetts Institute of Technology, Cambridge, MA 02139, USA*
[5]*Department of Civil and Environmental Engineering, Massachusetts Institute of Technology, Cambridge, MA 02139, USA*
[6]*Department of Psychological Sciences, University of Connecticut, Storrs, CT 06269, USA*
[7]*Kognia Sports Intelligence, Barcelona, Spain*
[8]*Laboratoire d'Hydrodynamique de l'École Polytechnique (LadHyX), Palaiseau, France*

[†]These authors contributed equally and are listed in alphabetical order
[‡]These authors contributed equally in the early development of the project and are listed in alphabetical order
[*]Corresponding author e-mail: skha89@mit.edu

S1 Further details on Methods

S1.1 Data pre-processing

As explained in the manuscript, we have used player and ball tracking data (25Hz) provided by the San Jose (SJ) Earthquakes collected by Second Spectrum (SS). The dribbles are encoded as individual entries on a table, where for each dribble we have information on the game, the ball handler (attacker), the defender and the minute and second (cents of second not available) when the event occurred.

The necessary pre-processing steps to fit the proposed model to the data consist of determining the start and end of dribble (from a discrete timestamp), as well as the initial velocities of the players involved in the dribble.

S1.1.1 Dribble start and end

We first need to precisely determine the span of the dribble, since event data only provides a discrete time, and approximate the velocity for both players at the start of the dribble, which are the input arguments to the model. Determining the span of the dribble is a highly subjective task, and we understand the approach we propose may have limitations.

Since each dribble is indexed by $t_0 = 60 \cdot \text{min} + \text{sec}$, where the second is rounded to an integer, we start by trimming the tracking data for both players for an interval of eleven seconds, namely $[t_0 - 1, t_0 + 10]$. Within this interval, the start of the dribble is set to the first frame where the attacker is within 50 cm of the ball and within 10 m of the defender. The end of the dribble is the first frame where the at least one of the following is satisfied: attacker is not within 1 m of the ball; the distance between defender and attacker is greater than 10 m.

There are some cases where an annotated event does not result in a valid dribble: (1) the attacker never comes within 50 cm of the ball and within 10 m of the defender during the eleven-second interval introduced above; (2) the attacker or defender do not have tracking data available; (3) the final duration of the dribble is less than 0.5 seconds. After applying these criteria, the final dataset encompasses 2714 dribbles.

S1.1.2 Evaluating player velocities with the Savitzky-Golay filter

Secondly, we need the two components of the players' velocities at the start of the dribble, and since SS only provides the magnitude of the velocity (and not its components), we use the framework below to extract meaningful velocities from the positional data.

To obtain player velocities, which are necessary to fit the model, we start from the player tracking data (x, y) sampled at discrete times t, the velocity fields (v_x, v_y) are computed with the following process [39]: Smooth player trajectories by applying Savitzky-Golay

(SG) filter, fit cubic splines, derive to obtain velocities, and smooth the obtained velocities with SG filter.

1. Apply a SG filter to x (resp. y) to smooth the signal, using a polynomial order 2 and a window size of 21 points, such that $\tilde{x} = SG(x)$ (resp. $\tilde{y}$).
2. Fit a cubic spline expansion to $\tilde{x}$ (resp. $\tilde{y}$), derive it and evaluate it on the discrete time values t, obtaining discrete velocities v_x (resp. v_y).
3. Apply a SG filter to v_x (resp. v_y) to smooth the signal, using a polynomial order 2 and a window size of 21 points, such that $\tilde{v}_x = SG(v_x)$ (resp. $\tilde{v}_y$).

The choice of polynomial order and window size is not arbitrary, but rather is the combination for which the discrete velocity values that we compute $\sqrt{\tilde{v}_x^2 + \tilde{v}_y^2}$ exhibit the highest (> 0.99) correlation with velocity magnitude data provided by Second Spectrum. It should be noted that the player velocities reported by SS exhibit physically impossible values on 0.02% out of the total tracking data frames, which is due to a limited accuracy in measuring the position of the players on those frames. We have ensured that the velocities computed to initialize the model are physically feasible for all the dribbles in our dataset.

S1.2 Parameter fitting

The optimization to find the combination of parameters that renders the best fit is performed using the COBYLA method available within Python's SciPy package. For each dribble, we input the real trajectories from attacker and defender as well as their initial velocities as described above, and then perform ten independent optimization runs using random parameter initialization. For each optimal parameter configuration we then evaluate the error in the player trajectories predicted by the model, and choose the parameter configuration that renders the lowest combined error of both trajectories. The error metric is computed for both the attacker ε_a and the defender ε_d using Equation (3) in the manuscript.

S1.3 Analysis of dribble error, distance and duration

After the dribbles have been pre-processed and fitted, we can investigate some basic stats on dribble distance, duration and fitting error. To that end, we show the univariate histograms and bivariate scatterplots and density plots for the (logscale) fitting error (attacker and defender), the traveled distance (attacker and defender) and the dribble duration in Fig. S1(a), along with the Pearson correlation coefficients. We see weak correlations between errors and distances traveled/dribble duration, meaning the model does not have a bias towards short or long dribbles (both in distance and duration). The errors on the attacker and defender trajectories are moderately positively correlated, and they both show Gaussian-like distributions. Finally, distance traveled by attacker and defender exhibit a

very strong correlation, as well as between both distances and dribble duration, due to the nature of how dribbles are defined in football.

S1.4 Dribble filtering criteria

In this section, we outline the relevant filters that have been employed throughout the study to ensure a meaningful interpretation of the model results.

S1.4.1 Distance traveled

We establish a minimum distance of 2 meters traveled by both players in order to accept the dribble. This choice is motivated by our hypothesis that the player dynamics that the model captures (e.g. attraction/repulsion to goal/opponent) are more salient for longer interactions, whereas they can be shadowed in situations where both players do not move far from their original location. The distance filter reduces the dataset to 1573 dribbles.

S1.4.2 Fitting error

For our analysis, we shall only consider dribbles where the model is able to accurately reproduce the players' trajectories, namely $\varepsilon_a, \varepsilon_d < 0.1$, meaning that we allow an average of 10% frame-by-frame deviation along the entire duration of the dribble. The error filter reduces the dataset to 1153 dribbles. Dribbles where the error exceeds the threshold can be viewed as scenarios where some of the limitations of the model become relevant, see Section S8.1.

S1.4.3 Parameter positivity

The premises of the dribbling model are that the attacker is attracted to the opposing goal and repelled from the defender, whereas the defender is attracted to both the own goal and the attacker. This behavior occurs when $\beta_a, \beta_d, \alpha_a, \alpha_d > 0$, see Section S8.2 for further information. Even though the optimization may render negative parameters, depicting more unusual situations such as an attacker being repelled from the opposing goal, for the statistical analysis we restrict to the category of dribbles where all parameters are positive, a total of 623 dribbles. Similar analyses may be carried out by accounting with the dribbles where one or more parameters are negative, but they were not pursued in this article due to the scarce number of dribbles in these other categories.

S1.5 Distance dependence of player parameters

As mentioned in the main text, we focus on dribbles with attacker-defender distance below 10 meters and assume that player parameters are nonzero constants in these situations. Fig. S1(b) confirms that player parameters do not show any significant distance dependence when the attacker and the defender are close to each other and validates our assumption.

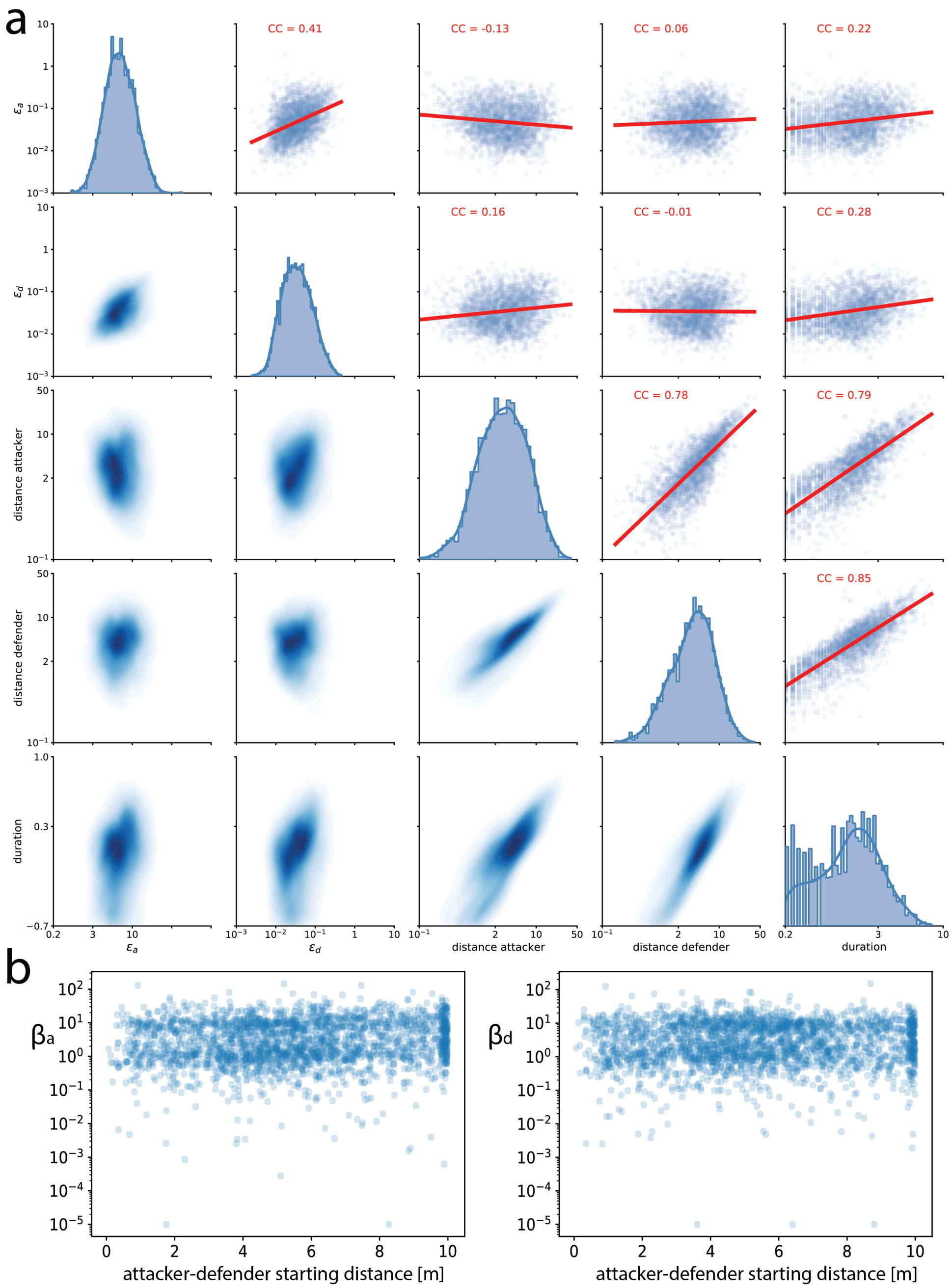

Figure S1: (a) Scatter plots, histograms, density plots and Pearson correlations between fitting errors, traveled distances and dribble duration, all variables shown in logscale. Correlation coefficients are computed with the log variables. (b) Scatter plots between β and the distance between the attacker and the defender at the start of the dribble.

S2 Stability of nonlinear system

In this section, we analyze the stability of the system (3) for an arbitrary potential. We distinguish the simplest formulation $n = 0$ that we use throughout this research, whereby there is no explicit distance dependence between the different agents, from the more generic formulations $n > 0$ that correspond to distance potentials in the form of $\propto r^{-n}$. To begin, we express the system as a first order system and in terms of both players' x and y coordinates.

$$\begin{aligned}
\dot{x}_a &= v_{xa} \, , \\
\dot{v}_{xa} &= -\frac{1}{\tau_a} v_{xa} + k_{ag} \frac{x_g - x_a}{\|\boldsymbol{x}_g - \boldsymbol{x}_a\|^{n+1}} - k_{ad} \frac{x_d - x_a}{\|\boldsymbol{x}_d - \boldsymbol{x}_a\|^{n+1}} \, , \\
\dot{y}_a &= v_{ya} \, , \\
\dot{v}_{ya} &= -\frac{1}{\tau_a} v_{ya} + k_{ag} \frac{y_g - y_a}{\|\boldsymbol{x}_g - \boldsymbol{x}_a\|^{n+1}} - k_{ad} \frac{y_d - y_a}{\|\boldsymbol{x}_d - \boldsymbol{x}_a\|^{n+1}} \, , \\
\dot{x}_d &= v_{xd} \, , \\
\dot{v}_{xd} &= -\frac{1}{\tau_d} v_{xd} + k_{dg} \frac{x_g - x_d}{\|\boldsymbol{x}_g - \boldsymbol{x}_d\|^{n+1}} + k_{da} \frac{x_a - x_d}{\|\boldsymbol{x}_a - \boldsymbol{x}_d\|^{n+1}} \, , \\
\dot{y}_d &= v_{yd} \, , \\
\dot{v}_{yd} &= -\frac{1}{\tau_d} v_{yd} + k_{dg} \frac{y_g - y_d}{\|\boldsymbol{x}_g - \boldsymbol{x}_d\|^{n+1}} + k_{da} \frac{y_a - y_d}{\|\boldsymbol{x}_a - \boldsymbol{x}_d\|^{n+1}}
\end{aligned}$$

The equilibrium points may be found by setting the temporal derivatives $\dot{x}$, $\dot{v}$ to zero, for which we can find that the system is at equilibrium if both players and the goal are in a straight line and the defender is located somewhere along the line between the goal and the attacker. Without loss of generality, for the equilibrium study we assume that all agents are located on the x-axis, and that $0 = x_g < x_d < x_a$, where x_a, x_d are upper-bounded by 100, which is the most common length of a football pitch. For each n, we establish the requirements of the player positions x_a, x_d and the attraction constants k_{ag}, k_{ad}, k_{dg}, k_{da} for equilibrium to be satisfied. Since the system above is nonlinear, we examine the eigenvalues of the Jacobian around each equilibrium point to determine the stability of the system. More specifically, for each combination of equilibrium states $0 < x_d < x_a \leq 100$, the system is stable if all eigenvalues have a negative real part and unstable if at least one eigenvalue has a positive real part. Furthermore, the system is oscillatory if there is at least a pair of eigenvalues with nonzero complex conjugate imaginary parts.

S2.1 Case $n = 0$

In this case, equilibrium is attained if the attraction constants satisfy $k_{ag} = k_{ad}$ and $k_{dg} = k_{da}$, regardless of the value of $x_a > x_d$

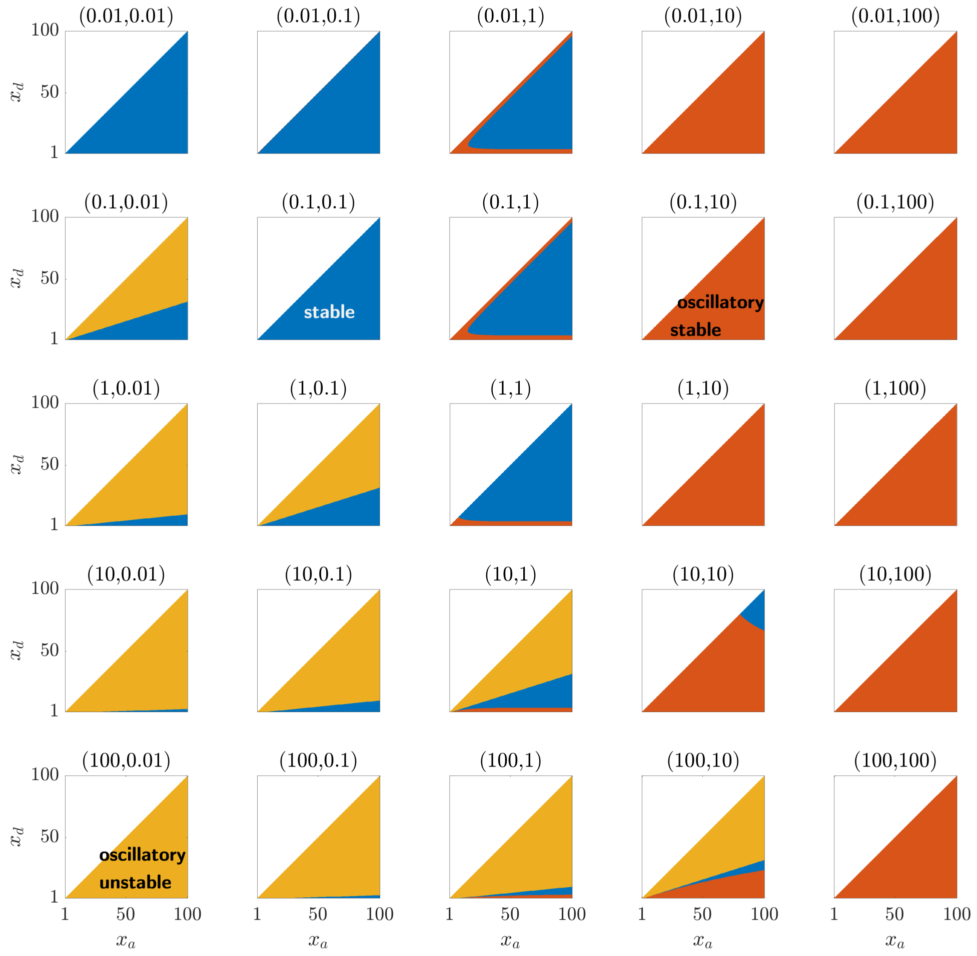

Figure S2: Stability plots for $n = 0$ and for different combinations of attraction constants, indexed in each subtitle as (k_{ag}, k_{dg}), computed in the vicinity of each equilibrium point. Blue and orange represent stable systems, whereas dark yellow represents an unstable system. Attacker position is shown on the x-axis and defender position is located on the y-axis, ensuring always that $x_d < x_a$ in order to satisfy equilibrium

Repeating this process for multiple combinations of attraction constants, while ensuring $k_{ag} = k_{ad}$ and $k_{dg} = k_{da}$, we compile the stability maps shown in Fig. S2. Each individual heatmap corresponds to a given value of the constants, denoted by (k_{ag}, k_{dg}) in the each subtitle, and since the position of the attacker is shown as the x-coordinate only the lower triangle of each image carries a physical interpretation, that is $x_d < x_a$. In terms of physical interpretation, the system remains stable (attacker not surpassing the defender) if the constants for the defender are larger than those of the attacker. Conversely, as the

defenders' constants shrink while the attackers' constants increase and the attacker comes closer to the goal, the system becomes unstable, signaling that the attacker beats the defender.

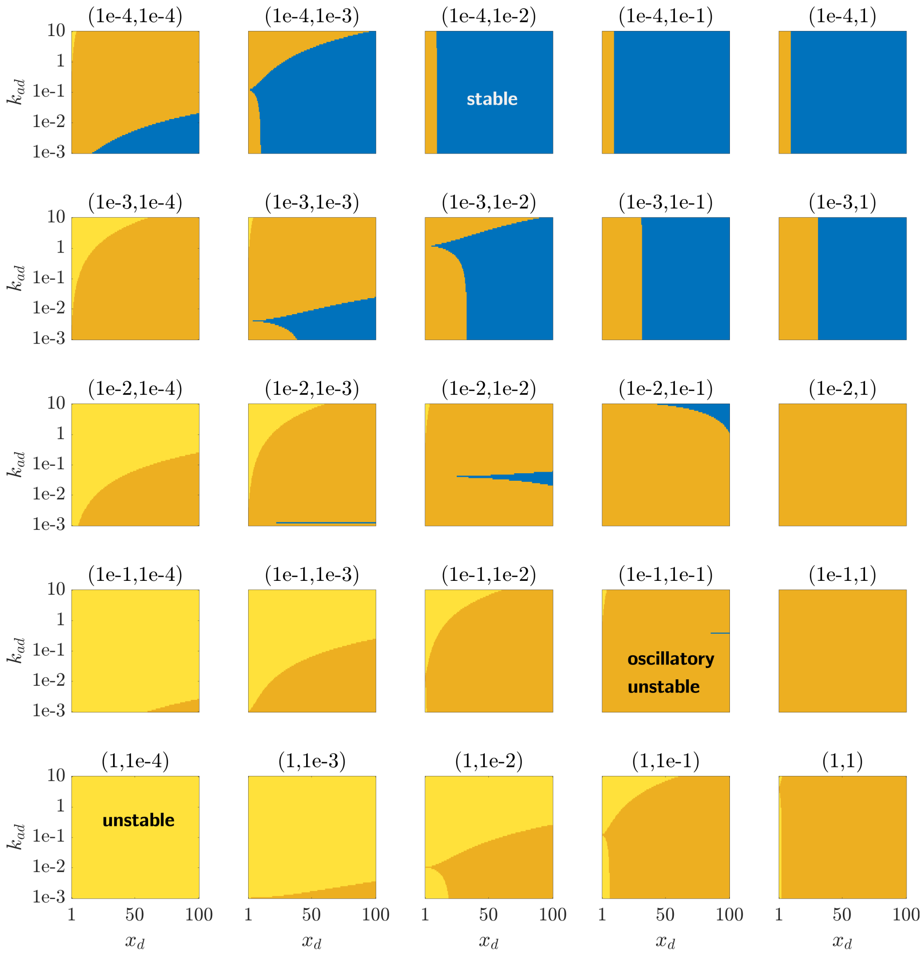

Figure S3: Stability plots for $n = 1$ and for different combinations of attraction constants, indexed in each subtitle as (k_{dg}, k_{da}), computed in the vicinity of each equilibrium point. Blue represents stable systems, whereas yellow represents an unstable system (dark yellow - oscillatory, light yellow - decaying unstable). Defender position is shown on the x-axis and k_{ad} is shown on the y-axis, whereasand k_{ag}, x_a are computed based on (S1) and (S2) such that equilibrium is satisfied.

S2.2 Case $n > 0$

In this case, equilibrium is attained if the attraction constants satisfy

$$\left(\frac{k_{ag}}{k_{ad}}\right)^{1/n} - \left(\frac{k_{dg}}{k_{da}}\right)^{1/n} = 1 \tag{S1}$$

and if the attacker's position is expressed as

$$x_a = x_d \left(\frac{k_{ag}k_{da}}{k_{ad}k_{dg}}\right)^{1/n} \tag{S2}$$

To numerically study stability, we first initialize the triad k_{ad}, k_{dg}, k_{da} and recover k_{ag} with (S1). We then set x_d between 0 and 100 and evaluate x_a with (S2). Repeating this process for multiple combinations of attraction constants we compile the stability maps shown in Fig. S2. Each individual heatmap corresponds to a given value of (k_{dg}, k_{da}) specified in the each subtitle, whereas the x-axis is used to display x_d and the y-axis to display k_{ad}. For ease of visualization we omit k_{ag}, x_a, which can be immediately obtained using (S1) and (S2). The results for $n = 1$ are shown in Fig. S3 and for $n = 2$ are shown in Fig. S4.

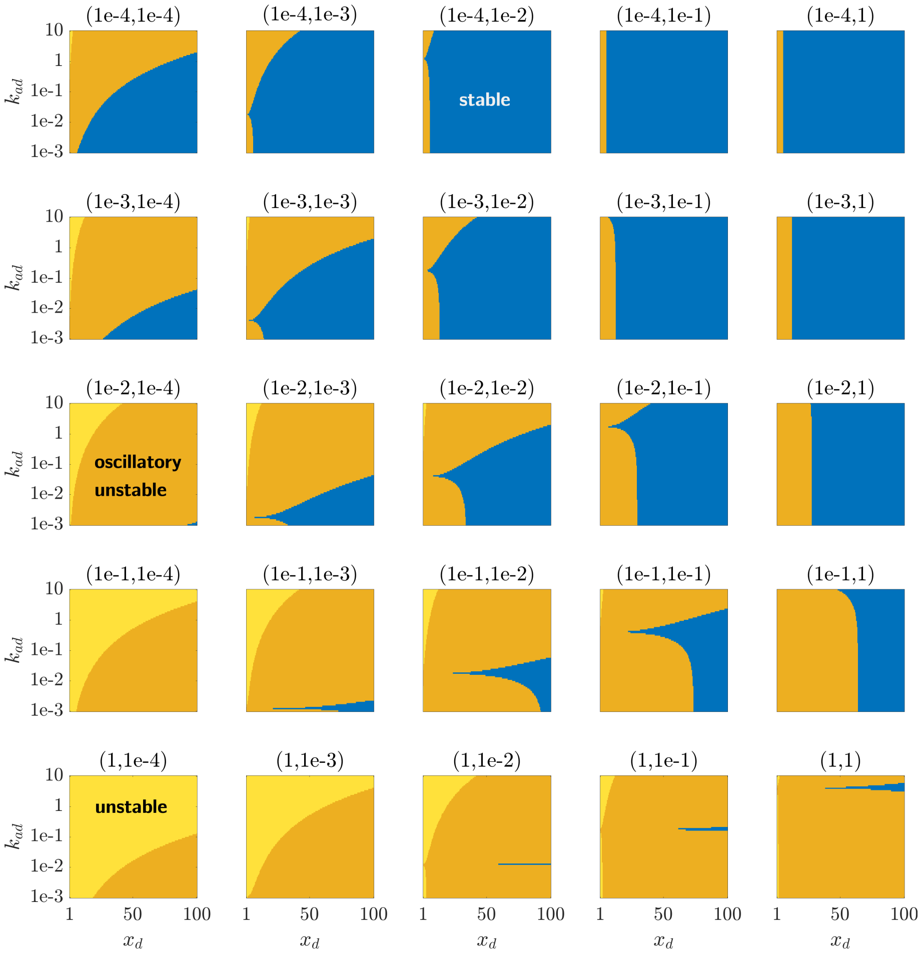

Figure S4: Stability plots for $n = 2$ and different combinations of attraction constants, indexed in each subtitle as (k_{dg}, k_{da}), computed in the vicinity of each equilibrium point. Blue represents stable systems, whereas yellow represents an unstable system (dark yellow - oscillatory, light yellow - decaying). Defender position is shown on the x-axis and k_{ad} is shown on the y-axis, whereas k_{ag}, x_a are computed based on (S1) and (S2) such that equilibrium is satisfied.

S3 Sensitivity analysis

The $\beta - \varepsilon$ distributions for both attacker and defender accounting for $\pm 25\%$ sensitivity are shown in Fig. S5, where we can observe that extreme values of β tend not to be sensitive. In addition, sensitive β values are clustered around 1, which is the value that defines a change in behavior, e.g. $\beta_a > 1$ indicates more attraction to goal than repulsion from opponent, and conversely for $\beta_a < 1$. Our interpretation is that dribbles where β matters are those where the dynamics of the players are influenced by both the opponent and the goal, whereas dribbles with extreme values of β one agent (either goal or opponent) heavily outweighs the other.

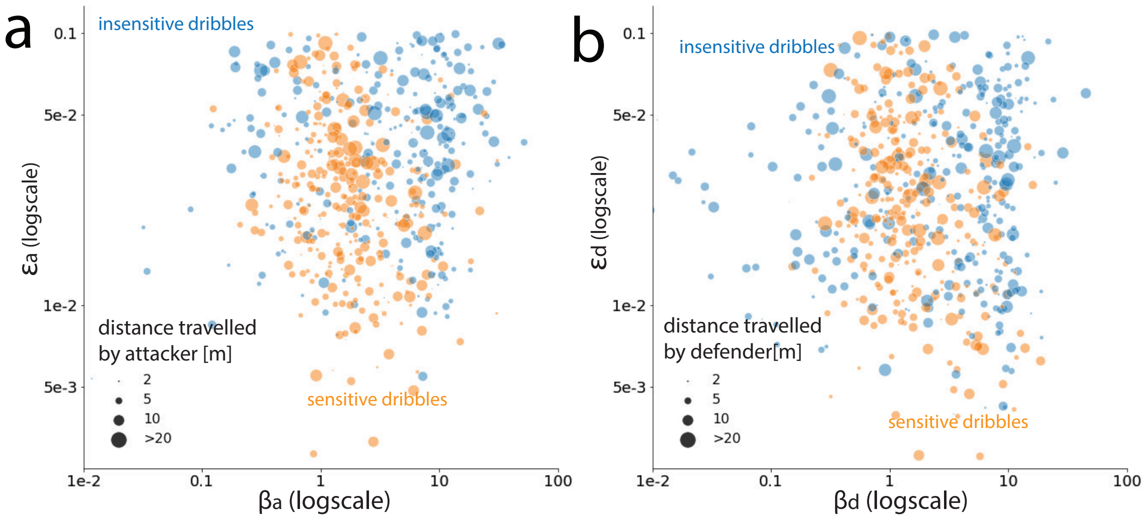

Figure S5: Visualization of sensitivity for all dribbles left after filtering by distance, error and positivity (total of 623). Size of dots corresponds to distance traveled by player, and orange dots are dribbles that are sensitive to a $\pm 25\%$ variation on the examined parameter (blue dots are insensitive dribbles). Note that despite spanning many orders of magnitude, β values corresponding to sensitive dribbles tend to be clustered around 1, which is where both attractive forces are balanced. a) Log depiction of β_a vs ε_a. b) Log depiction of β_d vs ε_d

Fig. S6 shows that relatively strong negative correlation (resp. weak positive correlation) exists between parameter sensitivity and player error metric (resp. player travel distance). Considering that the parameter sensitivity represents how much meaningful information the fitted parameter contains, these results indicate that those values obtained from well-fitted long dribbles should be more informative about player attributes, which agrees with our general intuition.

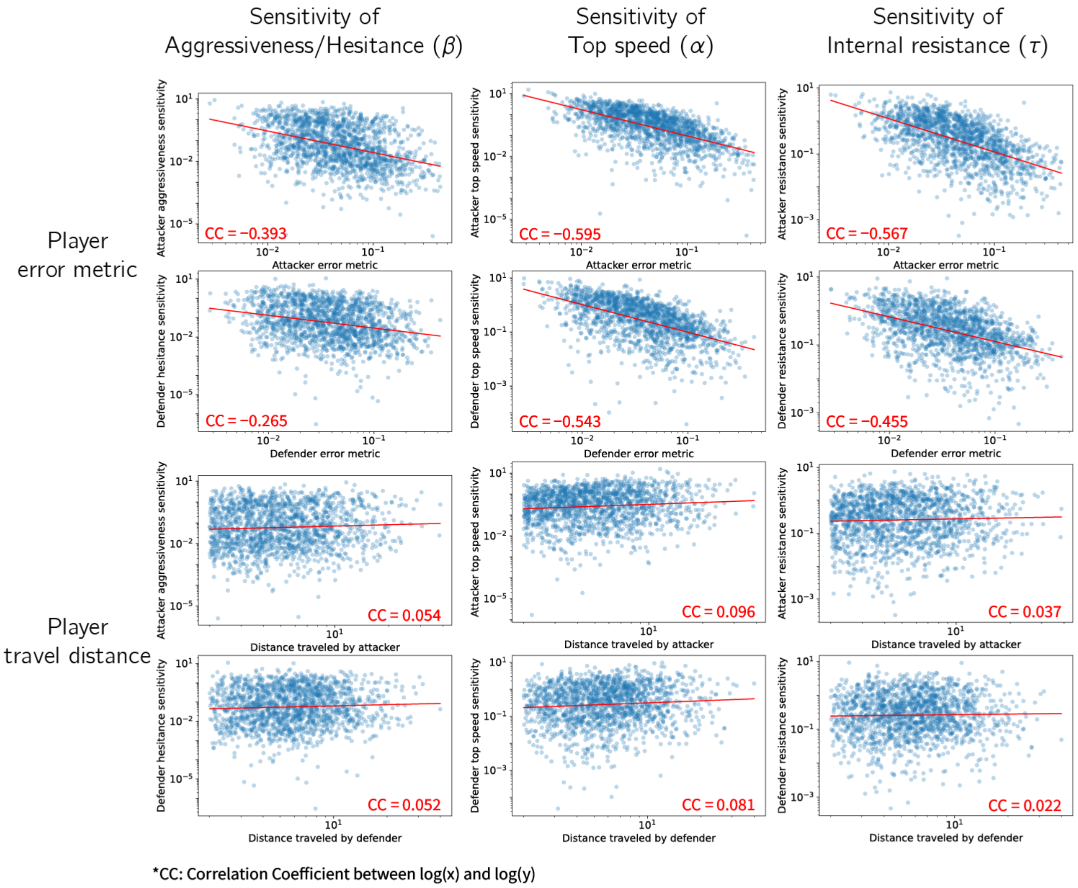

Figure S6: Scatter plots and Pearson correlations between player parameter sensitivities and fitting error/travel distance, all variables shown in logscale. Correlation coefficients are computed with the log variables

S4 Additional model validation examples

We showcase how the model effectively captures both the attacker and defender trajectories for several dribbling trajectories in Fig. S7 (active goal in yellow), where they overlap with the real tracking data from the players ($\varepsilon_a, \varepsilon_d < 0.01$).

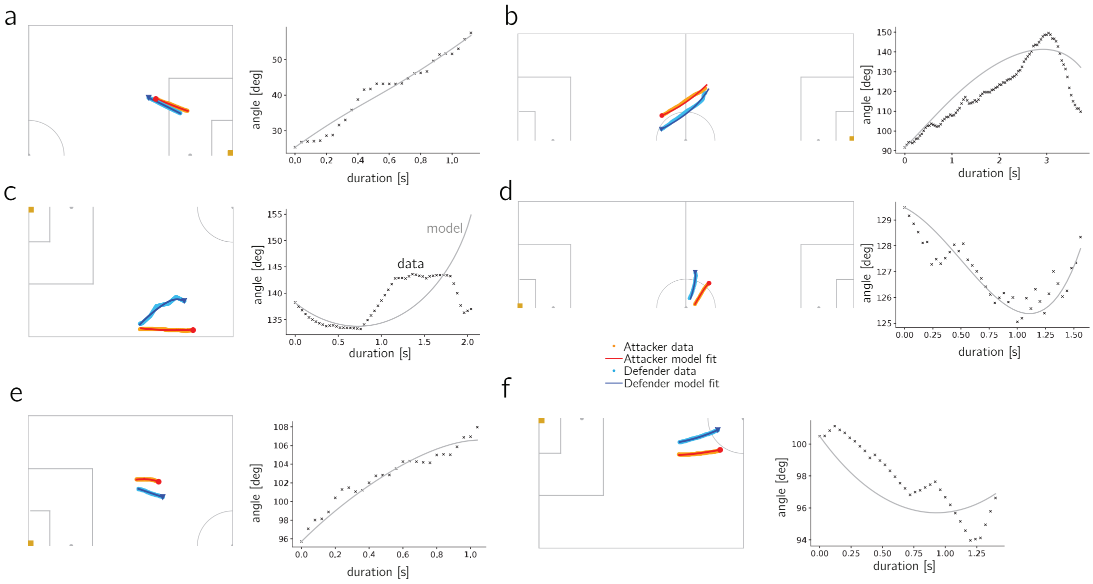

Figure S7: Six different situations comparing tracking data with model fit for trajectories and goal-defender-attacker angle. Legends are shared across subfigures.

S5 Alternative scoring methods

The dribble score proposed in the manuscript can be summarized as

$$z = \frac{1}{3}(z_\theta + z_{AG} + z_{AD}) \ ,$$

where the first term z_θ accounts for the change in attacker-defender-goal angle $\theta \in [0, \pi]$ (where $\theta = 0$ corresponds to dribbles with the attacker in front of the defender and both aligned with the goal and $\theta = \pi$ to the attacker behind the defender); the second term z_{AG} accounts for the relative change in attacker's distance to the goal; and the third term z_{AD} accounts for the distance between the attacker and defender at the end of the dribble, see Fig.3 for a sketch on how these quantities are evaluated.

As explained in the manuscript, for the angle and attacker-goal term we rely on the empirical cumulative distribution function of the dribble dataset (filtered by minimum distance), whereas for the attacker-defender term we rely on a rectified linear unit (ReLU) to extract the score. The value of the different scores on each individual dribble (in scatterplots), the univariate distributions of values and how they are converted into scores (via either empirical CDF or ReLU) is depicted in Fig. S8.

In this section, we discuss first the impact on z_{AD} (and consequently z) of the ReLU's distance cutoff δ, and then introduce two alternative scoring methods that might be used when rating the dribbles and evaluating the impact of parameter changes.

S5.1 Attacker-defender cutoff

The z_{AD} term is defined as a rectified linear unit with a δ cutoff, such that the score is 1 if the final attacker-defender distance $AD_{t=T}$ is above δ and varies linearly between 0 and 1 if $AD_{t=T} \in [0, \delta]$. The rationale behind this choice is that beyond a certain final attacker-defender distance the score of the dribble should remain the same. We propose a lower bound of $\delta > 1.5$m based on our football intuition (the minimum distance for an attacker to have successfully cleared the defender) and an upper bound of $\delta < 2.5$m, and ascertain that for δ in this range the trends of the predicted mean score with respect to parameter variations remain the same, albeit the actual score values change slightly, see Figs. S9 and S10. Since the recommendations of the model are robust to changes in δ, we choose $\delta = 2$m for the results in the manuscript.

S5.2 Without attacker-defender term

We have also considered the alternate score $z_2 = (z_\theta + z_{AG})/2$, that is without the attacker-defender term. The dribble scores computed with z_2 will exhibit larger variance than those with z, since they involve the summation of only two uniformly distributed variables (whereas z involves an additional non-uniform variable). The predicted mean score is also

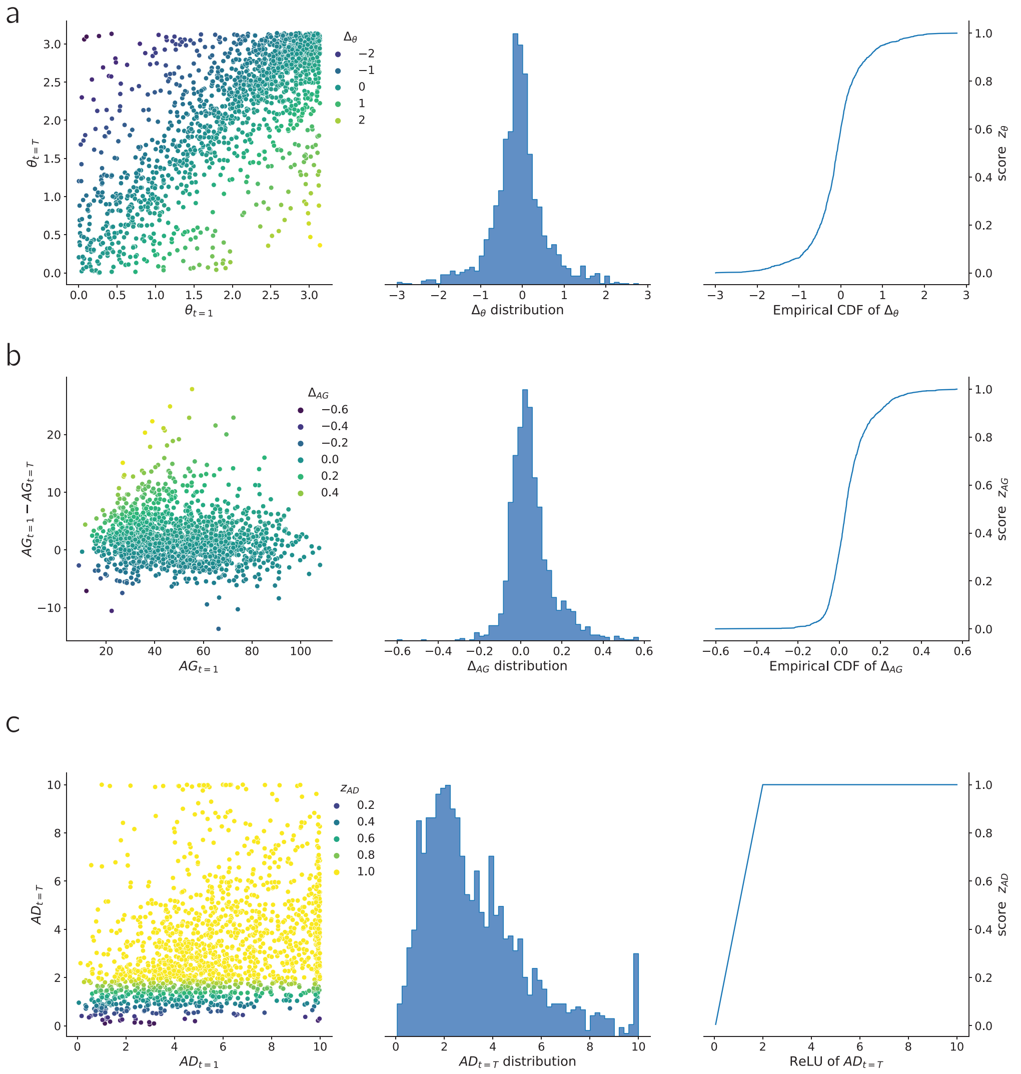

Figure S8: Information on individual score components, from left to right: (a) scatterplot of dribbles colored by value of angle difference $\Delta\theta$; distribution of angle difference; empirical CDF to obtain z_θ. (b) Scatterplot of dribbles colored by value of relative distance of attacker to goal Δ_{AG}; distribution of relative attacker-goal distance; empirical CDF to obtain z_{AG}. (c) Scatterplot of dribbles colored by final attacker-defender distance $AD_{t=T}$; distribution of relative final attacker-defender distance; rectified linear unit with cutoff 2m CDF to obtain z_{AD}.

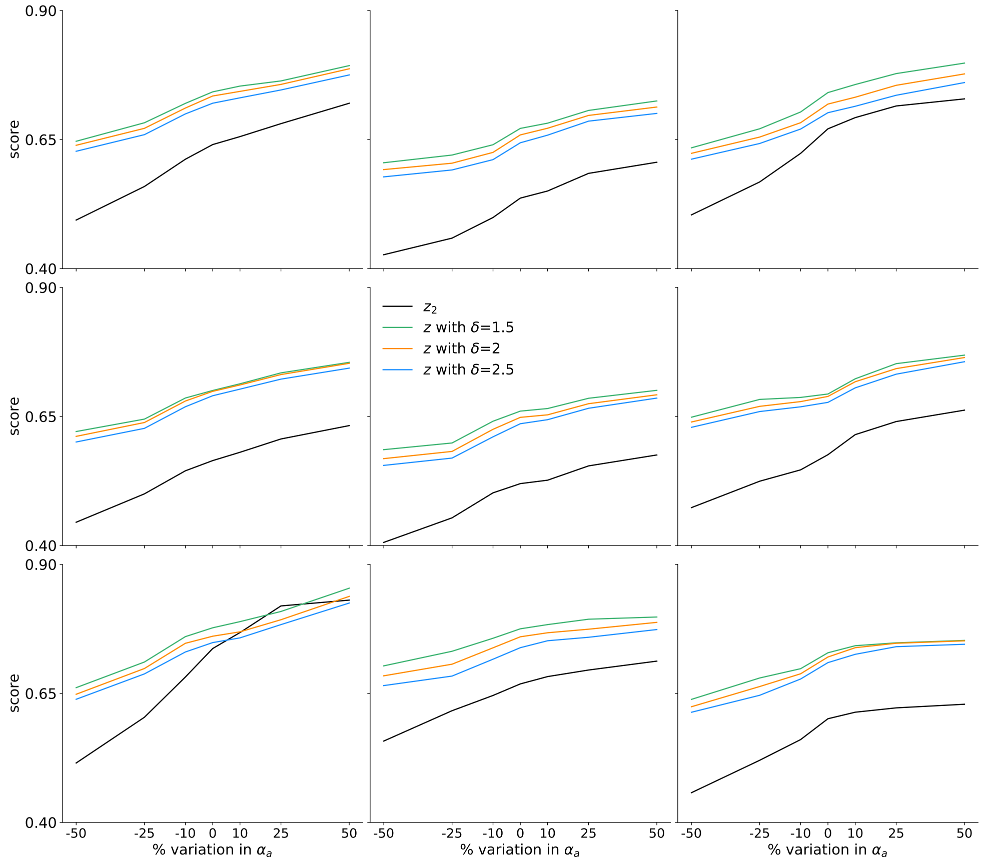

Figure S9: Predicted mean score of all dribbles computed with z_δ and z_2, where each panel corresponds to a different player, with respect to variations in α_a. Legend is shared across subfigures.

depicted in black in Figs. S9 and S10, where we see that for the majority of cases z_2 yields the same recommendations as z. Hence, we decided to use z instead of z_2, since z integrates the important notion of finishing a dribble not in close proximity of the defender that z_2 neglects.

S5.3 Binary classifier using Second Spectrum labels

In the events data provided by Second Spectrum there is an additional binary column for each 1v1 situation, namely `defender beaten`, that is 1 if the defender has been beaten and 0 otherwise. In order to leverage these labels, an alternative scoring criteria may be constructed based on a machine learning model trained upon the binary labels `defender beaten`. The main caveat with these labels is that we do not know how they have been

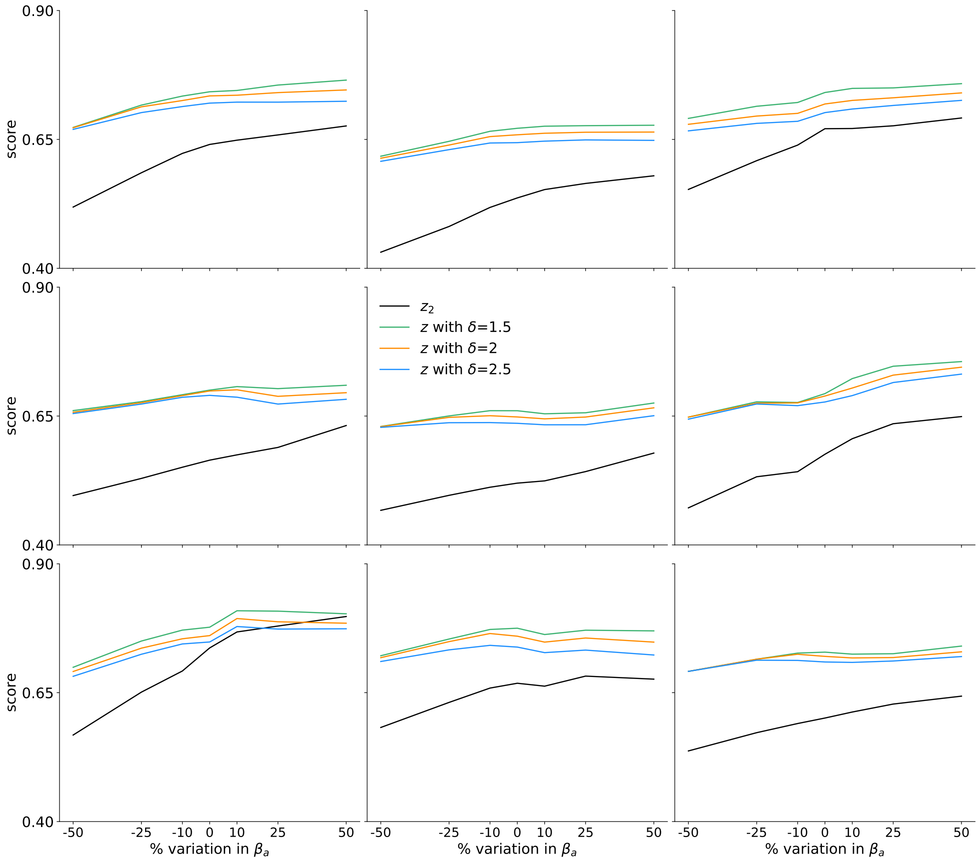

Figure S10: Predicted mean score of all dribbles computed with z_δ and z_2, where each panel corresponds to a different player, with respect to variations in β_a. Legend is shared across subfigures.

obtained, and in light of the impossibility to ascertain its accuracy (other than with manual inspection), we merely report the methodology proposed as if the labels were ground truth. That is the reason why we prefer the scoring introduced above than the SS labels for our manuscript results.

Starting with the training set of the 1573 dribbles filtered by distance (since the outcome should be independent of the fitting error), and first build a classifier using the initial and final angle θ, the initial and final attacker-goal distance and the final attacker-defender distance. We first balance the training set (66% of labels are 0) and find that a Gaussian Process (GP) classifier with a radial basis function kernel with unit length scale (the default classifier provided by the `sklearn` Python package) renders the best performance and the least amount of overfitting, namely 89% train and test accuracy and AUC $= 0.95$. If we

combine this circumstance with the fact that the GP classifier is well calibrated, we can use for each individual dribble the output GP probabilities as the probability of beating the defender.

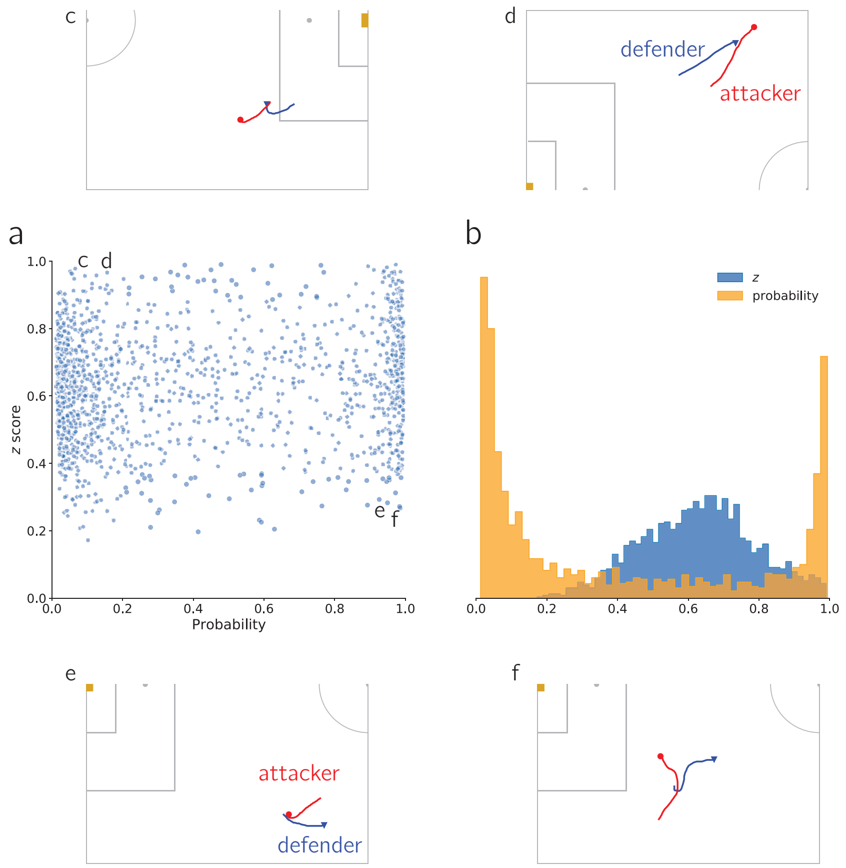

Figure S11: Comparison between scores z and probabilities computed with Gaussian Process on the dataset of 1573 dribbles filtered by traveled distance: (a) scatter plot of dribbles; (b) histogram of score and probability distributions; (c,d) examples of dribbles with high scores and low probabilities; (e,f) examples of dribbles with low scores and high probabilities.

Once the GP classifier is available, we may use it as a black-box model to predict the probability of beating the defender given the input features (initial and final angle θ, initial and final attacker-goal distance, final attacker-defender distance). Similarly as before, the impact of changes in either the behavioral or physical parameters can be analyzed at the player level, bearing in mind that the figure of merit is not the score of the dribble, but

the probability of beating the defender. The distribution of scores and probabilities for the 1573 dribbles in the dataset is shown in Fig. S11(a), where it is apparent that they measure two different quantities. We also show four examples of dribbles with high discrepancies between z and the GP probability, two with high scores and low probabilities in Figs. S11(c,d) (namely the attacker advancing towards the goal while the defender is still in front of him the entire duration of the dribble, hence not beaten), and two with low scores and high probabilities in Figs. S11(e,f) (the defender is no longer a threat since the attacker does not go towards the goal and defender does not follow, but rather guards own goal). Furthermore, the histogram of scores is unimodal since it is formed by the sum of three independent distributions (two of them uniform), whereas the histogram of probabilities is bimodal since the objective of the classifier is to maximize the certainty in assigning a label to each example, thus leaving the examples where the model is confused with probabilities around 0.5, see Fig. S11(b).

S6 Examples of impact of parameter variations on individual dribbles

Similarly to what we presented in Fig. 3 of the manuscript, we show two additional examples how changes in parameters impact individual dribbles, both at the trajectory and at the score level, see Fig. S12.

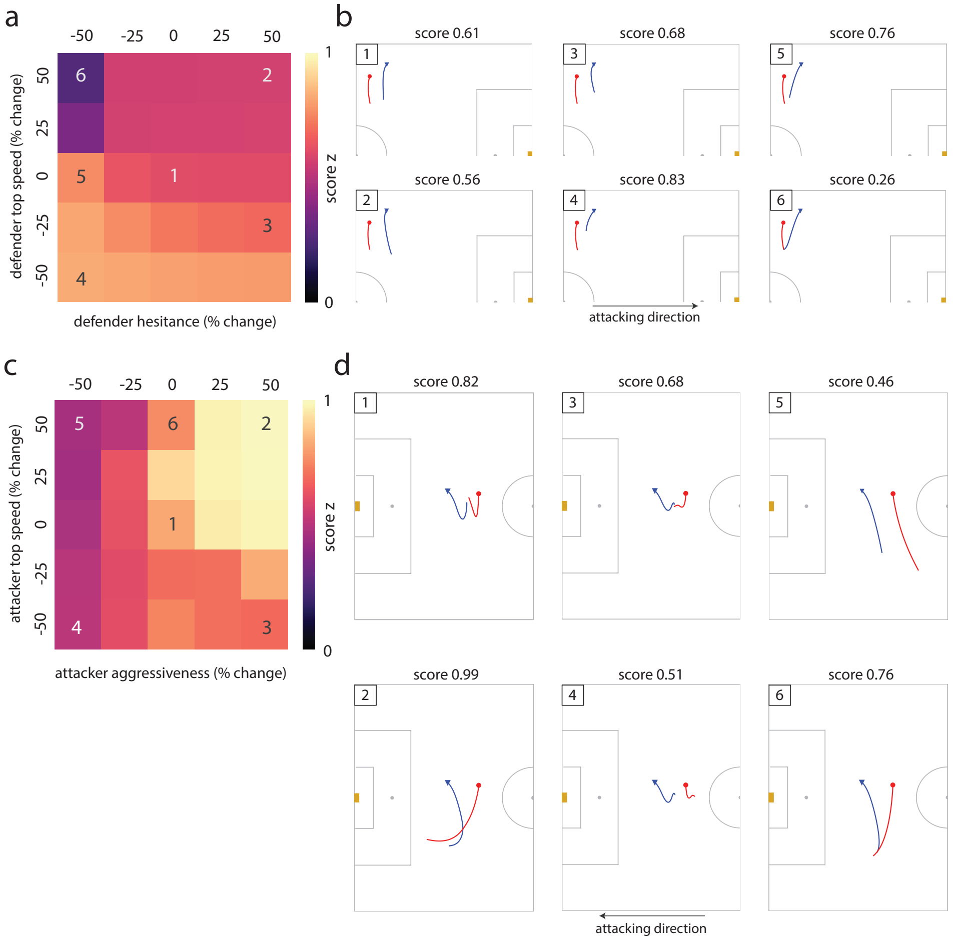

Figure S12: Impact of defender parameter variations (a,b) attacker parameter variations (c,d) on individual dribbles: (a) heatmap of predicted scores for simultaneous $\pm 25, \pm 50\%$ variations in defender hesitance β_d and defender top speed α_d; (b) simulated trajectories and predicted score for prescribed percentual change in behavioral parameters; (c) heatmap of predicted scores for simultaneous $\pm 25, \pm 50\%$ variations in attacker aggresiveness β_a and attacker top speed α_a; (d) simulated trajectories and predicted score for prescribed percentual change in top speed parameters.

S7 Impact of parameter changes in players' mean dribble score

In this section, we show the predicted mean score for variations of the parameters, both individual variations (line plots with 95% confidence interval) and combined (heatmaps showing just the mean score), for all the players in our dataset with more than 10 dribbles, see Fig. S13. It can be extracted that whereas all players benefit from a boost in top speed, only half of them actually experience advantages when increasing aggressiveness, suggesting it is a much more nuanced and player-dependent parameter (as expected). In terms of actual scores, players (a,g,h,i) exhibit the highest mean score in the absence of parameter changes and also the best perspectives in light of both increases and decreases in speed/aggressiveness, according to their heatmaps. Finally, we can single out (g) as the player with best projected scores due to parameter boosts, and conversely players (b,e) as examples of players that would significantly worsen their mean dribble scores as a result of reductions in aggressiveness or (particularly) top speed.

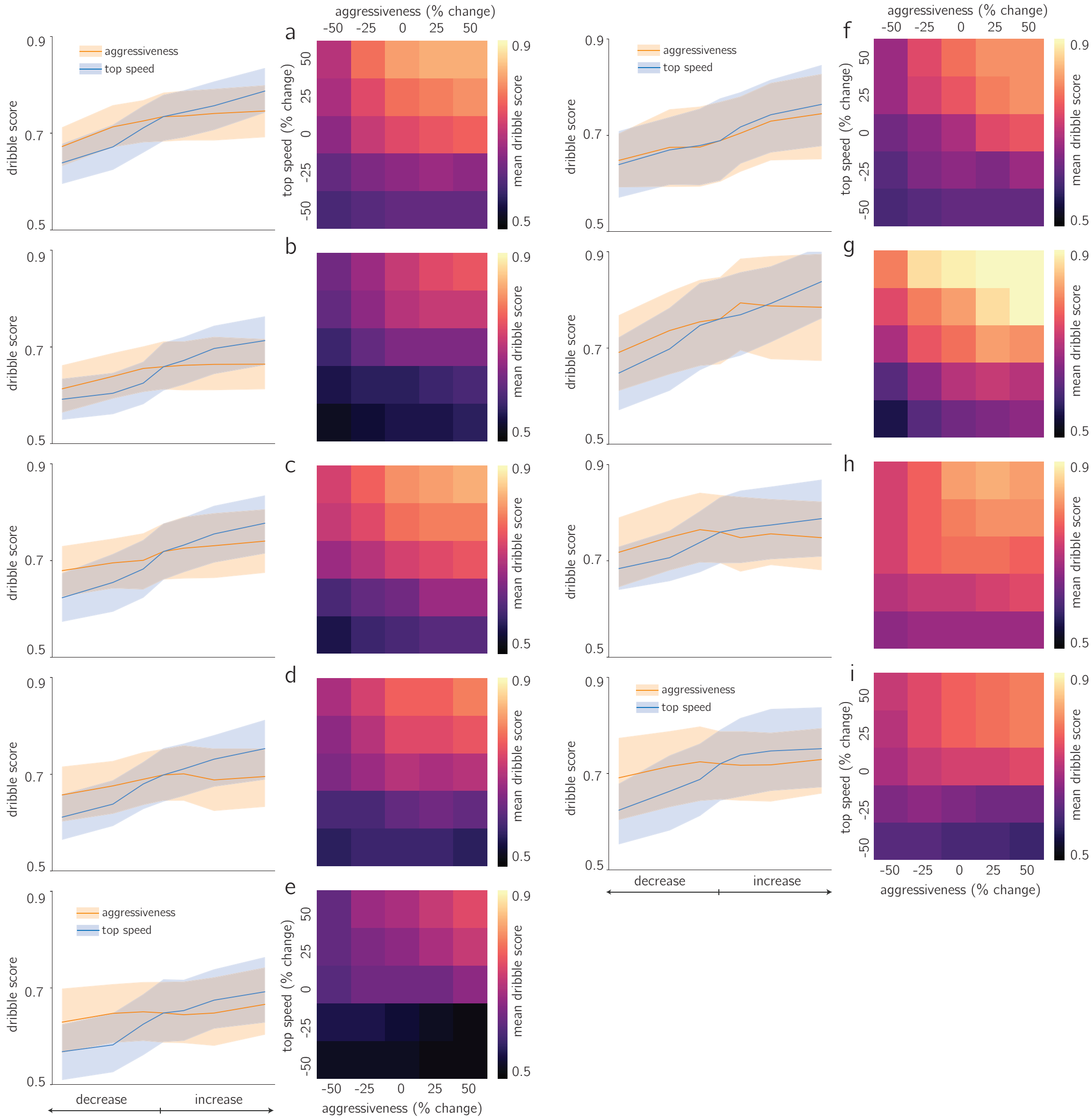

Figure S13: Is it more beneficial to improve top speed and/or aggressiveness? Each pair of line-heatmap corresponds to a player, indexed by letters (a-i). On the line plots, we show the predicted mean dribble score with 95% confidence interval when changing β_a, α_a independently; on the heatmaps, we show the predicted mean score (without confidence interval) when changing β_a and α_a simultaneously. Results suggest all players benefit from increase in top speed, whereas some players (a,c,e,f,g) also benefit from increasing aggressiveness (although speed has a stronger impact).

S8 Model limitations and potential improvements

S8.1 Fitting accuracy

Even though we showed that the proposed model enables meaningful analysis and provide valuable insights, it should be kept in mind that it assumes 1v1 situations to simplify the dribble dynamics. Thus, there exist several cases where the model exhibit limited accuracy, see Fig. S14. For example, since the model is based on differential equations and assumes smooth trajectories, it does not effectively capture the sudden change in player moving directions, see Fig. S14(a). We believe that the addition of random skill factors in the model equations may mitigate this problem. In addition, the model did not take the influence of sideline into account, see Fig. S14(b). In reality, players can be thought to experience repulsive force from the sideline to prevent losing possession. Adding repulsion from the sideline, especially when players are close to it, should further improve the model. Lastly, and most importantly, the model does not capture the players' behaviors when multiple attackers or defenders are involved. Fig. S14(c) shows the trajectories of the same dribble situation, but with two different defenders. Both of those two defenders significantly affect the attacker trajectory simultaneously, and it can be seen that assuming 1v1 situation with either defender does not fully explain the attacker's behavior. Expanding the model to incorporate multiple attackers and defenders may elucidate more complicated decision-making processes from players in wide variety of situations, and is therefore an interesting future research direction.

S8.2 Negative parameters

There exist several dribbles that exhibit negative α or β, which are not as intuitive considering the nature of attackers, who want to score, and defenders, who want to protect the goal. Due to the way β and α are defined, their sign depends on the underlying sign of the attraction/repulsion values, namely for the attacker:

$$\begin{aligned}
\beta_a > 0,\, \alpha_a > 0 &\Leftrightarrow k_{ag} > 0,\, k_{ad} > 0 \\
\beta_a > 0,\, \alpha_a < 0 &\Leftrightarrow k_{ag} < 0,\, k_{ad} < 0 \\
\beta_a < 0,\, \alpha_a > 0 &\Leftrightarrow k_{ag} < 0,\, k_{ad} > 0 \\
\beta_a < 0,\, \alpha_a < 0 &\Leftrightarrow k_{ag} > 0,\, k_{ad} < 0
\end{aligned}$$

and similarly for the defender:

$$\begin{aligned}
\beta_d > 0,\, \alpha_d > 0 &\Leftrightarrow k_{dg} > 0,\, k_{da} > 0 \\
\beta_d > 0,\, \alpha_d < 0 &\Leftrightarrow k_{dg} < 0,\, k_{da} < 0 \\
\beta_d < 0,\, \alpha_d > 0 &\Leftrightarrow k_{dg} < 0,\, k_{da} > 0 \\
\beta_d < 0,\, \alpha_d < 0 &\Leftrightarrow k_{dg} > 0,\, k_{da} < 0\ .
\end{aligned}$$

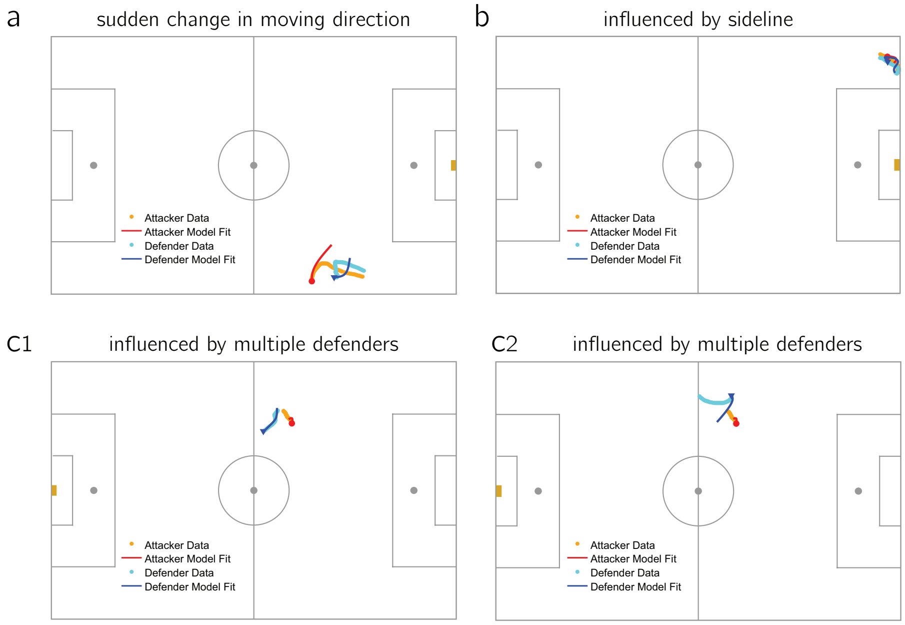

Figure S14: Demonstration of the current limitations of the model. The model exhibits limited accuracy when: (a) Players suddenly change moving directions. (b) Player trajectories are affected by sidelines. (c) Multiple attackers or defenders are involved in the dribble event.

Since the behavior of the players is dictated by the sign of the k values, using the above relations this behavior can be extracted by inspecting the signs of α, β. Thus, negative α and/or β indicate an opposite behavior from what we assumed when building the model (*e.g.* attacker going away from goal, defender not attracted to attacker). Through manual inspection, we found that those negative parameters are primarily coming from the influence of other player(s) who are not listed as the attacker or the closest defender. Fig. S15 shows example cases with negative α_a and β_a, where the attacker either dribbles toward the defender or away from the goal. It becomes evident from the trajectories that the closest defender is not the attacker's primary concern for those cases, and the attacker is actually trying to avoid the defender who is believed to be positioned between the attacker and the goal. Thus, we conclude that negative parameters do not reflect the actual players' decisions, but are in fact the artifact coming from the assumption of the model –1v1 dribble situation– and result from the dribbles where multiple attackers or defenders are simultaneously involved. As mentioned, expanding the model to incorporate multiple players can be a potential solution.

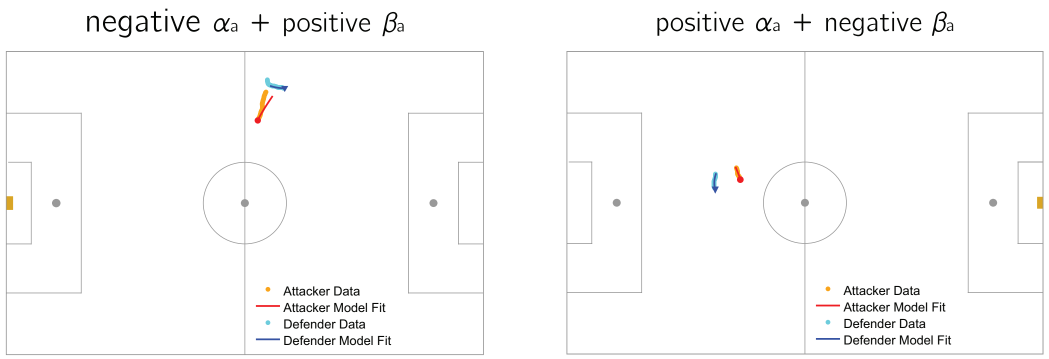

Figure S15: Example cases of negative player parameters.
